# Blocking ASIP to Protect MC1R Signaling and Mitigate Melanoma Risk: An In Silico Study

**DOI:** 10.3390/ph19010114

**Published:** 2026-01-08

**Authors:** Farah Maarfi, Mohammed Cherkaoui, Sana Afreen, Mohd Yasir Khan

**Affiliations:** 1 Department of Digital Engineering and Artificial Intelligence, College of Science, Long Island University, Brooklyn, NY 11201, USA; farah.maarfi@liu.edu (F.M.); mohammed.cherkaoui@liu.edu (M.C.); 2 Department of Food and Nutrition, Era University, Lucknow 226003, India; sana.23.era@gmail.com

**Keywords:** agouti signaling protein (ASIP), melanocortin-1 receptor (MC1R), pharmacophore, virtual screening, melanogenesis

## Abstract

**Background:** Melanin protects skin and hair from the effects of ultraviolet (UV) radiation damage, which contributes to all forms of skin cancer, including melanoma. Human melanocytes produce two main types of melanin: eumelanin provides effective photoprotection, and pheomelanin offers less protection against UV-induced skin damage. The agouti signaling protein (ASIP) antagonizes the melanocortin-1 receptor (MC1R), hinders melanocyte signaling, and shifts pigmentation toward pheomelanin, promoting UV vulnerability. In this study, we aim to discover compounds that inhibit ASIP–MC1R interaction and effectively preserve eumelanogenic signaling. **Methods:** The ASIP–MC1R interface-based pharmacophore model from ASIP is implicated in MC1R receptor protein engagement. We performed virtual screening with a validated pharmacophore model for ~4000 compounds curated from ZINCPharmer and applied drug-likeness filters, viz. ADMET and toxicity profiling tests. Further, the screened candidates were targeted for docking to the ASIP C-terminal domain corresponding to the MC1R-binding moiety. Top compounds underwent a 100-nanosecond (ns) run of molecular dynamics (MD) simulations to assess complex stability and persistence of key contacted residues. **Results:** Sequential triage, including pharmacophore, ADME–toxicity (ADMET), and docking/ΔG, yielded a focused group of candidates against ASIP antagonists with a favorable fit value. The MD run for 100 ns supported pose stability at the targeted pocket. Based on these predictions and analyses, compound ZINC14539068 was screened as a new potent inhibitor of ASIP to preserve α-MSH-mediated signaling of MC1R. **Conclusions:** Our in silico pipeline identifies ZINC14539068 as a potent inhibitor of ASIP at its C-terminal interface. This compound is predicted to disrupt ASIP–MC1R binding, thereby maintaining eumelanin-biased signaling. These findings motivate experimental validation in melanocytic models and in vivo studies to confirm pathway modulation and anti-melanoma potential.

## 1. Introduction

Melanocytes play a central role in conferring protection to the skin from the photocarcinogenic effects of solar ultraviolet exposure [1]. It is unequivocal that ultraviolet is the main etiological factor for skin cancers, including melanoma. Melanoma skin cancer remains a major global health concern. According to the American Cancer Society (ACS) statistical data for the year 2025, around 104,960 new cases of melanoma will be diagnosed in the United States. Of these melanoma cases, approximately 44,410 will occur in women and 60,550 in men [2]. By synthesizing the pigment melanin within specialized organelles, melanosomes, and transferring it to surrounding epidermal keratinocytes, melanocytes provide the skin with uniform pigmentation that reduces the genotoxic effects of UV rays. Human melanocytes, regardless of ethnicity or phototype, synthesize two main forms of melanin: pheomelanin, which is reddish-yellowish, and eumelanin, which is dark brown/black in color and has an efficient scavenging property to capture reactive oxygen species (ROS) [3].

Melanocortin 1 receptor (MC1R) and agouti signaling protein (ASIP) are key counter-regulators of cutaneous melanogenesis [4]. Under physiological stimulation by α-melanocyte-stimulating hormone (α-MSH), MC1R activates cAMP signaling and biases pigment synthesis toward eumelanin, which reflects superior ultraviolet (UV) photoprotection and antioxidant capacity [5,6]. The genomic structure of *MC1R* is quite complex, as it displays several splice variants and a high degree of polymorphism. It comprises four exons and yields several transcripts. The major transcript, with a 951 nucleotide coding region (ID ENST00000555147, named MC1R-001), contains exons 2, 3, and 4, with retention of two unspliced intervening sequences between exons 2 to 3 and 3 to 4. This transcript encodes for a 317-amino-acid integral membrane protein with all the structural characteristics of a family A GPCR. Expressed predominantly on melanocytes, this protein is related to human pigmentary as well as non-pigmentary functions, including DNA repair [7]. Upon stimulation by α-MSH, adrenocorticotropic hormone, 12-O-tetradecanoylphorbol-13-acetate, or the synthetic analog [Nle4, DPhe7]-α-MSH, MC1R activates Gs/adenylate cyclase, elevating intracellular cAMP. cAMP signaling upregulates melanogenic enzymes tyrosinase (TYR), TYRP1, and TYRP2. This biases the pigment synthesis toward photoprotective, antioxidant eumelanin, thereby providing cells with photoprotective and antioxidant activities [8,9].

Conversely, when α-MSH is absent or at a very reduced level, ASIP endogenously antagonizes MC1R, diminishing cAMP and tyrosinase activity. This shifts melanogenesis toward pheomelanin, leading to less UV protection and promoting ROS generation and DNA damage, thereby increasing melanoma risk susceptibility [10,11,12,13]. ASIP is a cysteine-rich peptide of 132 amino acids encoded by the *ASIP* gene located on chromosome 20q11.2 and functions as a competitive inhibitor of MC1R. Although ASIP shares no primary sequence similarity with α-MSH, it binds MC1R with comparable affinity and effectively blocks α-MSH-driven receptor activation and downstream signaling [14]. As an endogenous antagonist and inverse agonist of MC1R, ASIP suppresses MC1R-mediated cAMP responses in human melanocytes and thereby governs the eumelanin–pheomelanin switch at the receptor–ligand interface.

The biological mechanism of ASIP provides a rationale for a non-classical therapeutic strategy that targets ASIP rather than relying on exogenous MC1R agonists that directly force receptor activation [15,16]. Inhibiting ASIP removes endogenous antagonistic restraint on MC1R, allowing restoration of physiological α-MSH–MC1R signaling in a more regulated and potentially selective manner. Importantly, human genetic studies implicate variants near the *ASIP* locus in pigmentation phenotypes and melanoma susceptibility, supporting the translational relevance of modulating this pathway. Collectively, these observations establish ASIP as a mechanistically grounded intervention point that directly controls MC1R pathway output and pigmentation biology, justifying its selection as the primary target in this study.

ASIP is known to comprise three functional loops: N-terminal, active, and C-terminal.

The N-terminal loop of ASIP is primarily known to involve secretion, stability, and accessory protein interactions and does not directly contribute to MC1R engagement [17].

The active loop comprises a conserved RFF amino acid (Arg-Phe-Phe, 117–119 amino acids) motif, which partially engages the MC1R within the transmembrane pocket. This contact is seen to be the same as that for α-MSH, indicating that RFF of ASIP mimics the HFRW sequence (His–Phe–Arg–Trp, positions 6–9) of α-MSH [18]. The HFRW is the main pharmacophore of α-MSH essential for MC1R activation. An in vitro study demonstrated that the cysteine-rich C-terminal domain of ASIP is sufficient for full MC1R antagonism and can bind melanocortin receptors with equal or greater affinity when compared to the full-length sequence. Alanine scanning identifies the RFF triplet as an essential segment for antagonist activity [19]. Convergent chimera and modeling studies also confirm that the C-terminal loop is necessary for high-affinity binding and inverse agonism. The deletion of the C-terminal region reduces the binding affinity of ASIP towards MC1R by 10-fold. Misplacement of this loop can flip peptides from antagonists to agonists (increases cAMP signaling), indicating that precise loop geometry is required to stabilize the inactive receptor state [16]. The C-terminal VLSLN loop (Val–Leu–Ser–Leu–Asn; residues 129–133) of ASIP is involved in hydrophobic interactions with the putative residue Arg109 of MC1R located within the LVARAA (Leu–Val–Ala–Arg–Ala–Ala; residues 106–111) region. Additionally, a salt bridge formation between Asp109 of ASIP and Arg109 of MC1R further stabilizes the complex. This electrostatic interaction highlights the mechanistic features consistent with the C-terminus being both necessary and sufficient for ASIP’s modulatory effect on MC1R [14].

**Figure 1:**
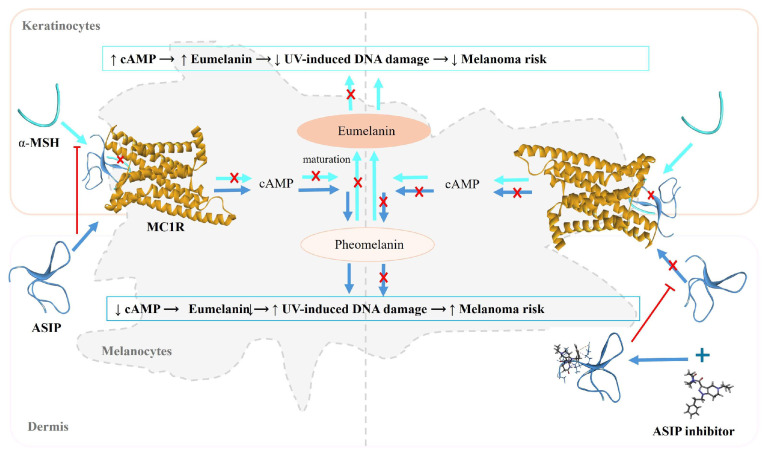
**Regulation of melanin synthesis via MC1R-α-MSH signaling and ASIP inhibition**: This schematic illustrates the role of α-MSH (cyan) and ASIP (blue) in modulating melanogenesis through the melanocortin 1 receptor (MC1R) (gold) in skin. Keratinocytes (epidermis) produce α-MSH, which binds to MC1R on melanocytes (basal layer), activating a signaling cascade that increases cAMP and promotes eumelanin synthesis, leading to reduced UV-induced DNA damage and lower melanoma risk. In contrast, ASIP, secreted by dermal fibroblasts, antagonizes MC1R, reducing cAMP levels and shifting pigment production toward pheomelanin, which is less protective and associated with increased melanoma risk. The figure also shows the action of an ASIP inhibitor, which blocks ASIP binding to MC1R, thereby restoring α-MSH-mediated signaling and promoting eumelanin synthesis. (Arrows indicate activation or signal flow; red T-shaped bars indicate inhibition; red “X” symbols indicate blocked or suppressed interactions; and the “+” symbol denotes pharmacological inhibition of ASIP).

Since the C-terminal region of ASIP provides a valid experimental scaffold for this study, we utilized the NMR solution structure (PDB ID: 2KZA), which includes ASIP residues 80–132. This short fragment is responsible for MC1R recognition and antagonism. The 2KZA structure accurately represents the “disulfide stapled knotted architecture” with a few stabilizing substitutions (Pro103Ala, Pro105Ala, Gln115Tyr, and Ser124Tyr) and is supported by peer-reviewed experimental studies, making it a reliable template for small molecule designing. Mechanistically, by focusing the docking around the VLSLN loop of the ASIP C-terminus with a small molecule, we intentionally disrupt the ligand orientation and misalign the RFF active loop (Arg117-Phe118-Phe119), which is critical for antagonizing MC1R. This reduces ASIP’s ability to engage the α-MSH (His–Phe–Arg–Trp, positions 6–9) binding on MC1R [16]. This whole illustration is well summarized in Figure 1.

## 2. Results

### 2.1. ASIP Binding Pocket Interaction with MC1R

The stereochemical quality of the selected ASIP protein (PDB ID:2KZA) was also assessed using a Ramachandran plot generated in BIOVIA Discovery Studio and presented in Figure S1. The plot shows that the majority of residues occupy favored and additionally allowed regions, with the observed distribution reflecting the intrinsic flexibility of the NMR-derived ASIP structure. No significant backbone stereochemical issues were observed. Therefore, the selected ASIP structure was further used for the investigation of different molecular compound identification and interaction processes.

In interaction studies, not all residues of protein are involved in protein–protein and protein–ligand interactions, making the identification of binding residues a critical step in molecular interaction studies. According to previous research, the C-terminal region of ASIP plays a critical role in its function as an inverse agonist by forming a contact point with the extracellular loop 1 (ECL1) of MC1R [14,20].

**Figure 2:**
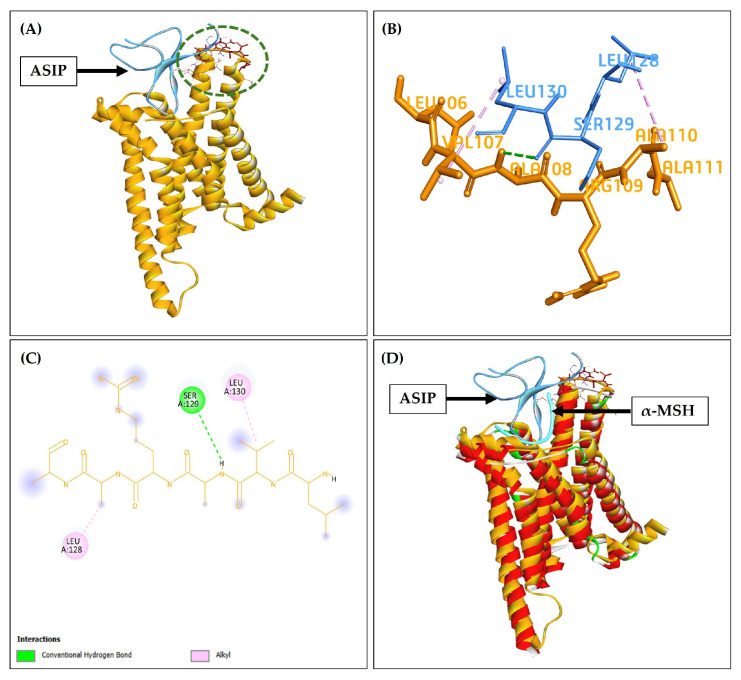
**ASIP and MC1R interaction**: (**A**) ASIP (blue) embedded in the MC1R (the green dotted circle represents the ASIP-MC1R interaction). (**B**) Interaction between residue Leu128, Ser129, and Leu130 of ASIP (blue) and MC1R residues included Leu106, Val107, Ala108, Arg109, Ala110, and Ala111 (golden). (**C**) A 2D diagram showing the ASIP docking pose with the binding site of MC1R. The dotted green lines and dotted pink lines represent conventional hydrogen bonding and alkyl hydrophobic interactions. (**D**) Superimposition of interacted ASIP_MC1R with α-MSH (cyan)_MC1R on each other.

To investigate this interaction, we focused on the LVARAA (MC1R EC1 106–111; Leu–Val–Ala–Arg–Ala–Ala) and VLSLN (ASIP C-terminal loop; Val–Leu–Ser–Leu–Asn) motifs of MC1R and ASIP, respectively, aiming to determine whether these motifs indeed interact within the same pocket of the protein MC1R as that used by α-MSH. To perform interaction between ASIP and MC1R, the proteins were prepared, followed by energy minimization, and protein–protein docking was performed using the ZDOCK method of Biovia (BIOVIA Discovery Studio v24.1.0.321712). In our study, ASIP was found to interact with MC1R through the same pocket/residues reported earlier [14], shown in Figure 2A, suggesting a conserved mode of binding. The ZDOCK score was 16.7 for the top-ranked pose of ASIP-MC1R. The 3D and 2D interaction interface residues identified for ASIP were Leu128, Ser129, and Leu130, while those for MC1R included Leu106, Val107, Ala108, Arg109, Ala110, and Ala111 (Figure 2B,C).

Structural superimposition of the ASIP–MC1R complex with the α-MSH–MC1R complex revealed that both ASIP and α-MSH occupy an overlapping binding pocket on MC1R (Figure 2D). This spatial overlap supports a competitive binding mechanism, consistent with previous reports describing ASIP as an antagonist of MC1R [21]. While the present docking analysis did not directly assess functional signaling outcomes, the observed pocket overlap provides a structural rationale for how ASIP binding may sterically hinder α-MSH engagement at its native receptor site [16]. Because ASIP engages MC1R through a defined C-terminal interface that overlaps with the α-MSH binding pocket [22], the ASIP C-terminal interaction surface was adopted as a receptor-based pharmacophoric template. Targeting this interface is expected to disrupt ASIP_MC1R complex formation by competing for key binding determinants, rather than mimicking α-MSH agonism. Our study is totally focused on a structure-based hypothesis rather than a functional validation of MC1R signaling modulation.

### 2.2. Pharmacophore Model Generated Based on ASIP’s Interaction with MC1R

**Figure 3:**
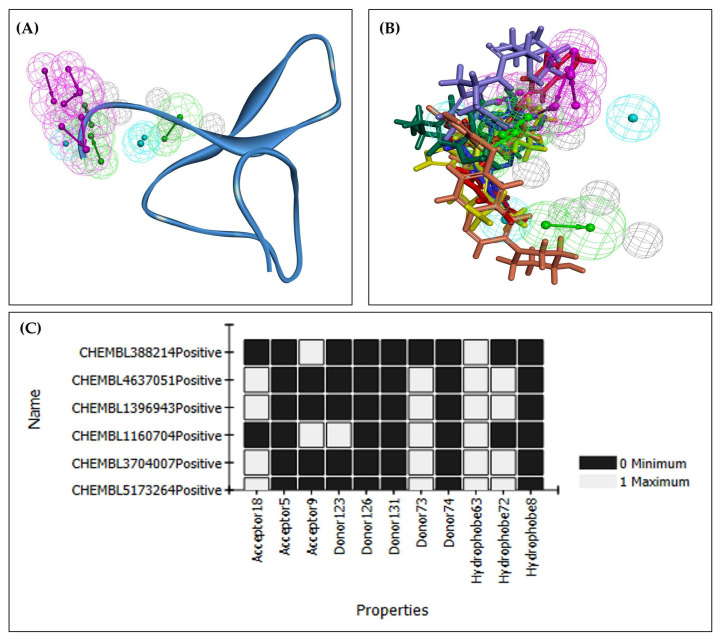
**Pharmacophore model**. (**A**) ASIP with pharmacophore interaction map showing 11 features: hydrogen bond acceptors (HBAs) (green mesh), hydrogen bond donors (HBDs) (magenta mesh), and hydrophobes (HYs) (cyan mesh). (**B**) Pharmacophore mapping of putative active compounds CHEMBL5173264 (yellow), CHEMBL3704007 (brown), CHEMBL1160704 (dark green), CHEMBL1396943 (red), CHEMBL388214 (purple), and CHEMBL4637051 (blue). (**C**) Heat map representing the pharmacophore features mapped by active-like compounds.

A pharmacophore describes the three-dimensional spatial and electronic arrangement of essential features (e.g., hydrogen bond acceptors (HBAs), hydrogen bond donors (HBDs), and hydrophobes (HYs)) needed for optimal ligand–receptor interactions. In this study, the pharmacophore model based on ASIP’s interaction with MC1R was generated, focusing specifically on the VLSLN region of ASIP. The generated model consisted of HBA, HBD, and HY features located within the receptor’s active site sphere. Since multiple vectored features may represent a single interaction point with an active site residue, only a reasonable subset of features was selected for virtual screening. Selection criteria included the orientation of feature vectors pointing within a 2.00 Å radius of the putative binding site (VLSLN), ensuring spatial relevance to the core interaction region. Based on these protein-derived interaction sites, a total of 11 pharmacophoric features were identified and incorporated into the final model presented in Figure 3A, which was further validated.

### 2.3. Validation of ASIP Based Pharmacophore Model

The goal of pharmacophore validation was to evaluate the ability of the generated model to correctly identify active-like ligands while minimizing false positives. In the absence of experimentally confirmed ASIP ligands, a curated validation dataset of 18 compounds was assembled, consisting of 10 docking-enriched putative actives and 8 kinase inhibitors used as putative inactive controls with no known or predicted interaction with ASIP.

Out of the 10 putative active ligands, 6 were successfully mapped to the pharmacophore model (Figure 3B), whereas none of the 8 putative inactives showed any mapping. The validation metrics were calculated as follows:

Sensitivity (Recall for Putative actives) = True Positives/(True Positives + False Negatives) = 6/10 = 0.60

Specificity (Recall for Putative inactives) = True Negatives/(True Negatives + False Positives) = 8/8 = 1.00

Enrichment Factor (EF) at 6 hits = (TP/total hits)/(Total actives/total dataset) = (6/6)/(10/18) = 1/0.555 ≈ 1.8

These results indicate that the pharmacophore model correctly recovered 60% of the docking-enriched putative actives, with no false positives among the kinase inhibitor controls, and identified active-like ligands nearly twofold better than random selection. Additionally, the fit value, which evaluates the geometric alignment between a ligand and the pharmacophore, was calculated for each compound. Higher fit values reflect a better structural match. The LibDock scores for the 10 putative active ligands, together with their fit values and mapped pharmacophore features, are summarized in Table 1.

Among these, CHEMBL5173264 exhibited the highest fit value and mapped multiple key features (Acceptor18, Donor73, Hydrophobe63, and Hydrophobe72), as shown in Table 1, providing the most complete pharmacophoric coverage and stable spatial alignment among the mapped ligands. It was therefore selected as the reference ligand for subsequent virtual screening. A heat map (Figure 3C) was generated to visualize which pharmacophore features were preferentially mapped by the active-like ligands. According to Discovery Studio’s mapping protocol, a ligand must satisfy at least four pharmacophore features to be considered a hit. In our model, Hydrophobe63 was defined as a required feature, and at least one feature was required from the VLSLN C-terminal pocket group. For analysis, 11 representative pharmacophore features were selected. All six mapped ligands satisfied at least four of these features, including Hydrophobe63, confirming consistent interaction with the predicted VLSLN binding pocket.

Taken together, this internal, docking-based validation supports the reliability and specificity of the ASIP pharmacophore model. Although sensitivity is moderate, the high specificity and enrichment over random justify the use of this pharmacophore as a primary filter in large-scale virtual screening for novel ASIP modulators.

### 2.4. Pharmacokinetic and Toxicity Evaluation

Pharmacokinetic and toxicity evaluation of the screened ligands was carried out using the ADMET Descriptors and TOPKAT modules in BIOVIA Discovery Studio v24.1.0.321712 [23]. About 1803 ligands were subjected to ADMET profiling based on six critical parameters to assess their drug-likeness. Human intestinal absorption (HIA) was predicted using the AlogP98 vs. PSA_2D model, and ligands falling within Levels 0 and 1 (indicating good to moderate absorption) were retained. These compounds were further evaluated for aqueous solubility using a regression-based model, with ligands exhibiting acceptable solubility (logS between −6.0 and −2.0, Levels 2 to 4) selected. Blood–brain barrier (BBB) penetration was assessed based on logBB values, and ligands within Levels 1 to 3 were considered acceptable. CYP2D6 inhibition was predicted using a Bayesian model, and compounds with scores below 0.161 were classified as non-inhibitors to reduce potential drug–drug interaction risks. Hepatotoxicity predictions, based on SAR models, filtered out compounds likely to cause liver toxicity, while plasma protein binding (PPB) profiling deprioritized ligands with predicted binding greater than or equal to 90%, retaining those with low to moderate binding for improved bioavailability. After applying ADMET filters, a total of 97 ligands shown in Tables S1 and S2 demonstrated favorable pharmacokinetic properties. The 97 ligands that passed ADMET properties were further evaluated for TOPKAT toxicity profiling, including weight of evidence (WOE) for carcinogenicity prediction of compounds, AMES prediction for mutagenicity, skin irritancy, and skin sensitization prediction (Table 2). Based on TOPKAT mutagenicity and carcinogenicity profiling, 37 ligands were selected as drug-like candidates for downstream in silico investigations, including molecular docking. The outcome validates the reliability of the pharmacophore-based screening pipeline and highlights promising leads for the inhibition of ASIP–MC1R by binding with ASIP.

### 2.5. ASIP Docking with ADMET Pass Ligands

Docking of the 37 ligands that passed ADMET and toxicity tests was performed into the VLSLN-centered ASIP binding site. Initial ranking was performed using the −CDOCKER interaction energy score (−CDOCK score), where more positive values indicate stronger predicted binding affinity toward the ASIP binding pocket. To further refine ligand prioritization, binding free energy (ΔG) calculations were performed, and a consensus ranking strategy was applied.

All 37 ligands successfully docked within the ASIP pocket, indicating compatibility with the defined C-terminal interface. To strengthen confidence in ligand ranking and reduce scoring bias, the top-scoring ligands were further evaluated using LibDock from BIOVIA (BIOVIA Discovery Studio v24.1.0.321712) and AutoDock Vina (v1.1.2, Windows) as independent validation methods. Based on the combined binding scores and binding free energy/affinity, five ligands—ZINC14539068, ZINC24890597, ZINC12212035, ZINC64926416, and ZINC91321775—were selected for detailed analysis and pharmacophore mapping (Table 3).

Among these, ZINC14539068 demonstrated the most favorable overall profile, exhibiting a high −CDOCK score (35.38), the most negative binding energy from the MM-GBSA calculation (dG = −65.88 kcal/mol), and the highest pharmacophore fit value (1.91654), indicating optimal spatial alignment with the ASIP-derived pharmacophore model. The compound ZINC24890597 displayed a −CDOCK score of 28.31 and a pre-MDS MM-GBSA binding energy (dG) of −63.38 kcal/mol, together with a moderate fit value (1.71863), suggesting high binding affinity and acceptable pharmacophore alignment. ZINC12212035 also showed strong performance, with a −CDOCK score of 25.29, a binding energy (dG) of −60.59 kcal/mol, and a low fit value (1.40281), supporting less effective pharmacophore feature coverage. In contrast, ZINC64926416, despite showing a substantial −CDOCK score (27.22), exhibited a comparatively weaker binding energy (dG = −25.03 kcal/mol) and a lower fit value, indicating reduced complex stability and suboptimal pharmacophoric correspondence. ZINC91321775 showed a −CDOCK score of 29.26 and a weak binding affinity (dG = −15.61 kcal/mol), along with the lowest fit value (0.0037683), suggesting poor spatial alignment with the pharmacophore model and minimal feature matching.

**Figure 4:**
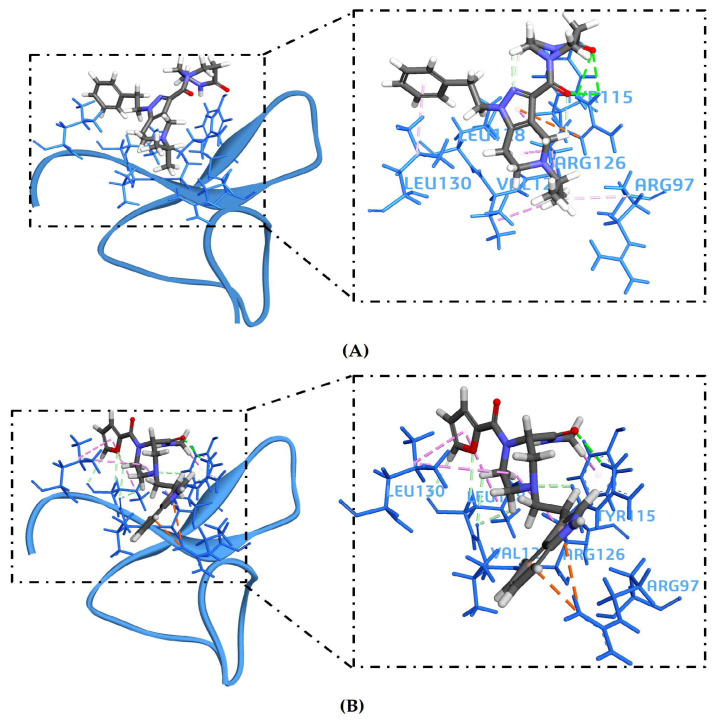
**Molecular docking and interaction analysis of top ASIP-binding Ligand-1** (ZINC14539068) and Ligand-2 (ZINC24890597). (**A**) Panels show the ZINC14539068 binding within the ASIP VLSLN-centered site and 3D interactions with key residues, including ARG97, TYR115, ARG126, ALA127, and LEU130. (**B**) Panels show the ZINC24890597 binding within the ASIP VLSLN-centered site and 3D interactions with key residues, including ARG97, TYR115, ARG126, ALA127, and LEU130.

Additionally, LibDock and AutoDock analyses further supported the selection of ZINC14539068 and ZINC24890597 (Table 3). ZINC14539068 achieved a favorable LibDock score (37) and a strong AutoDock-predicted binding affinity (ΔG = −5.8 kcal/mol), while ZINC24890597 showed the highest LibDock score (38) and a comparable AutoDock binding affinity (ΔG = −5.7 kcal/mol). The consistent high ranking of these two ligands across all scoring functions confirms their stable binding orientation and favorable ligand–receptor complementarity. Analysis of shortlisted ligands for their docked poses revealed that all compounds occupy a common region within the ASIP binding pocket corresponding to the C-terminal VLSLN interface implicated in MC1R recognition (Figure S2). The docked complexes of ligands ZINC14539068 and ZINC24890597 consistently engaged key ASIP residues, including Arg97, Tyr115, Arg126, Ala127, Ser129, and Leu130, and adopted similar spatial orientations (Figure 4). The two-dimensional (2D) interaction analysis of ZINC14539068 and ZINC24890597 (Figure S3) revealed that ZINC14539068 forms conventional hydrogen bonds with Arg126 and Tyr115, with additional stabilization arising from van der Waals and alkyl/π–alkyl interactions involving Arg97, Val127, Leu128, and Leu130. These interacting residues are located within the ASIP C-terminal VLSLN region critical for MC1R recognition, indicating that ZINC14539068 directly engages the core ASIP interaction interface through a balanced combination of polar and hydrophobic contacts. In comparison, ZINC24890597 displays a distinct yet overlapping interaction pattern within the ASIP binding pocket. This ZINC24890597 ligand forms a conventional hydrogen bond with Arg126, complemented by carbon–hydrogen bonding and stabilizing π–cation interactions with Arg97. Additional van der Waals and alkyl/π–alkyl contacts with Val127, Leu128, Leu130, and Tyr115 further contribute to hydrophobic complementarity within the C-terminal VLSLN interface. Overall, the convergence of −CDOCK scores, pre-MD MM-GBSA (dG), LibDock score, AutoDock binding affinity (ΔG), and pharmacophore fit values consistently supports the selection of ZINC14539068 and ZINC24890597 as the top candidates, emerging as strong inhibitory compounds.

**Figure 5:**
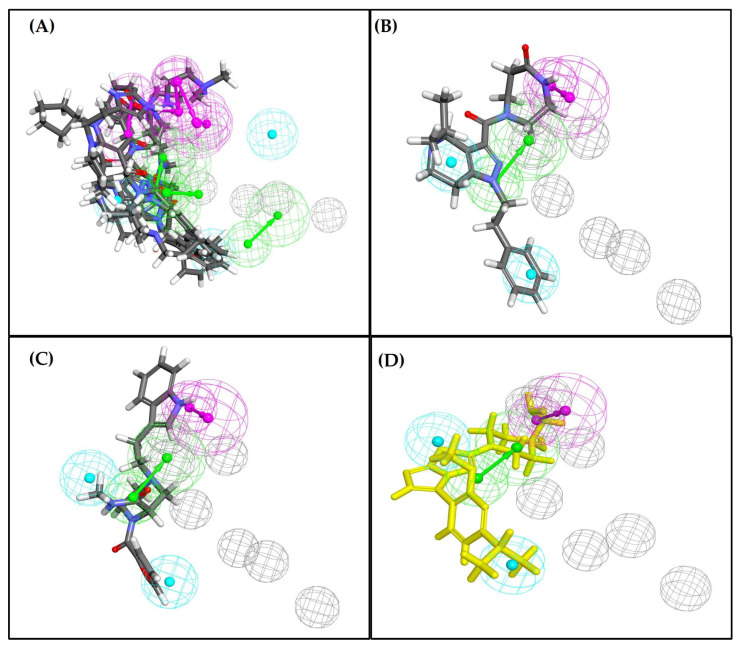
**Pharmacophore mapping of ASIP-binding ligands**. (**A**) The top five docked ligands exhibited substantial overlap with the predefined ASIP-derived pharmacophore model. (**B**,**C**) Pharmacophore mapping of ZINC14539068 and ZINC24890597 showed strong spatial alignment with the 3D pharmacophore features, including hydrogen bond acceptors/donors, hydrophobic regions, and aromatic rings. (**D**) Pharmacophore feature of CHEMBL5173264 (yellow), used as the reference for virtual screening via ZINCPharmer web server (v1.0, accessed September 2025).

Pharmacophore mapping analysis indicated that all five shortlisted ligands exhibit appreciable spatial overlap with the predefined ASIP-derived pharmacophore model (Figure 5A), reflecting conserved engagement of key binding features. Among these, ZINC14539068 (Figure 5B) demonstrated the highest fit value and the most complete alignment with the essential pharmacophoric features. ZINC24890597 (Figure 5C) also showed meaningful feature overlap, although with a comparatively lower fit value. Collectively, these results identify ZINC14539068 and ZINC24890597 as the two ligands that most closely reproduce the spatial and chemical characteristics of the validated ASIP pharmacophore model, closely mirroring the feature distribution observed for the reference compound CHEMBL5173264 (Figure 5D).

These two compounds—ZINC14539068 (Ligand-1) and ZINC24890597 (Ligand-2)—demonstrated superior binding stability, optimal pharmacophore alignment, and consistent ranking across multiple independent computational methods, justifying their prioritization for further structural and dynamic evaluation.

### 2.6. Dynamic Stability of the Protein–Ligand Complexes

RMSD analysis was performed over 100 ns simulations to assess the structural stability of ASIP in complex with the selected ligands ZINC14539068 as Ligand-1 and ZINC24890597 as Ligand-2, as compared to ASIP alone. The ZINC14539068–ASIP complex (Ligand-1–ASIP) showed gradual deviation between 5 and 13 ns, stabilizing around an average of 3 Å after 13 ns for the entire simulation period, indicating intrinsic flexibility in the absence of a bound ligand. However, the ZINC24890597–ASIP complex (Ligand-2–ASIP) showed a higher average RMSD of around 4 Å compared to both the native ASIP and Ligand-1–ASIP.

**Figure 6:**
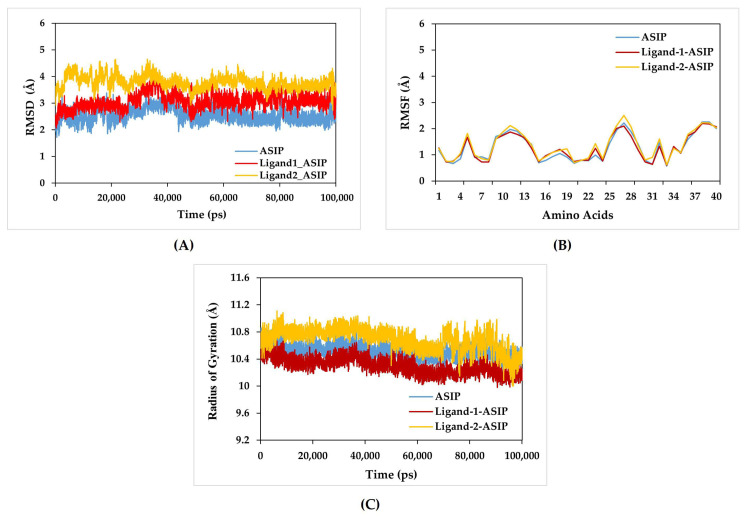
**The molecular dynamics simulation results of the ASIP and complex of ASIP with selected Ligand-1 and Ligand-2 compounds (Ligand-1–ASIP and Ligand-2–ASIP)**: (**A**) RMSD of native ASIP (ASIP) and complexes of Ligand-1 and Ligand-2 with ASIP. (**B**) RMSF of ASIP and complexes of Ligand-1 and Ligand-2 with ASIP. (**C**) RG of native ASIP and complexes of ASIP with Ligand-1 and Ligand-2.

In contrast, the native ASIP stabilized more rapidly, reaching equilibrium near 2.8 Å by ~4 ns. The ASIP system demonstrated RMSD fluctuations for 36–40 ns, followed by stabilization indicative of stable complex formation. Ligand-1–ASIP maintained a stable trajectory with RMSD around 2.2–3.5 Å, which is similar to the RMSD of the native ASIP structure without ligand (Figure 6A).

The RMSF analysis revealed that the residue-level flexibility patterns of the candidate ligand–protein complexes closely resembled those of the native ASIP, indicating that the ligand binding at the active site is both tight and stable. The predicted RMSF graphs of the C-α atom of native ASIP, as well as Ligand-1–ASIP and Ligand-2–ASIP, were plotted against the interacted residues based on the trajectory period (100 ns) of the MD simulation. The residues in the ligand–ASIP complex fluctuated around 0.5–2.5 Å in the 100 ns simulation time scale for Ligand-1–ASIP and Ligand-2–ASIP complexes (Figure 6B). The RMSF values for the amino acids of the Ligand-1–ASIP complex were close to the RMSF of ASIP but notably lower than the Ligand-2–ASIP complex.

To assess the global compactness of the protein during dynamic simulations, the RG was monitored over 100 ns. The detailed assessment of the RG and its directional components throughout the molecular dynamic simulation is shown in Figure 6C. Furthermore, the average RG value of the Ligand-1–ASIP complex was 10.4 Å, which is in close agreement with the average RG value of the ASIP at 10.5 Å (Figure 6C). However, the RG of Ligand-2–ASIP was around 10.7 Å, which is far from the native ASIP RG during a 100 ns molecular dynamics simulation. As a result of RG, it can be observed that the Ligand-1–ASIP complex exhibited relatively similar behavior of compactness and consistent values of RG as ASIP without ligand (native ASIP). Therefore, the protein–ligand complexes exhibited comparable and stable compactness throughout the 100 ns simulation period. A pronounced and consistent contraction along the gyration axis was observed for the Ligand-1–ASIP complex, suggesting that the complex adopts a stable and spatially constrained conformation under solvated simulated conditions. To assess the binding affinity of the Ligand-1 and Ligand-2 interaction with ASIP, their binding energy within the solvent environment was determined throughout the MD simulation using the MM-GBSA method. The findings revealed that the Ligand-1–ASIP complex exhibited the lowest binding free energy (−103 ± 4 kcal/mol), followed by the Ligand-2–ASIP (−91 ± 3 kcal/mol).

### 2.7. Hydrogen Bonds and Their Distance–Complex Stability

**Figure 7:**
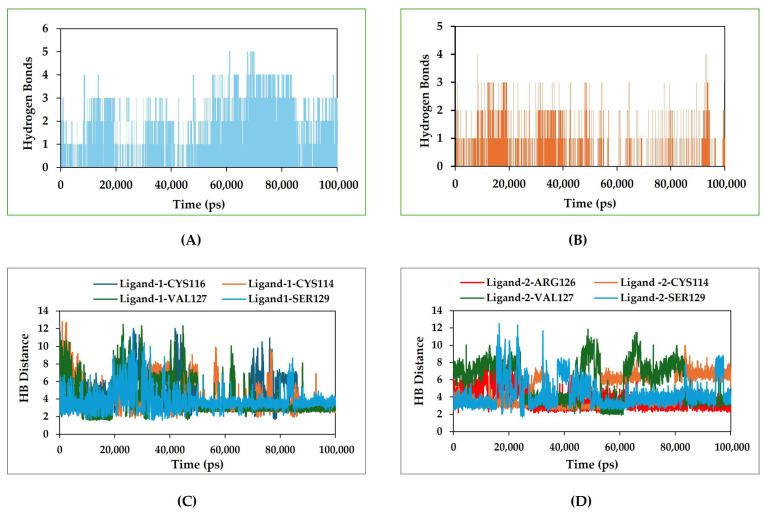
Number of hydrogen bonds established and the ligand–protein bond distance variations shown in plots generated from the 50 ns MD simulation trajectory of the ligand–ASIP complex. (**A**) Number of hydrogen bonds established between the ASIP and Ligand-1. (**B**) Number of hydrogen bonds established between the ASIP and Ligand-2. (**C**) Hydrogen bond distance variation for Ligand-1 and the interacting amino acid residues from ASIP. (**D**) Hydrogen bond distance variation for Ligand-2 and the interacting amino acid residues from ASIP.

To investigate the stability of the ligand interaction with protein, the measurement of intermolecular hydrogen bond development and its distance between the ligand and protein complex was evaluated. The complexes of Ligand-1 (ZINC14539068) with ASIP (Ligand-1–ASIP) protein maintained two to three hydrogen bonds throughout the entire simulation time (Figure 7A). However, the complexes of Ligand-2 (ZINC24890597) with ASIP (Ligand-2–ASIP) protein maintained only two hydrogen bonds throughout the entire 100 ns simulation time (Figure 7B).

Therefore, our molecular dynamics simulations indicated that the candidate Ligand-1 consistently maintained two to three hydrogen bonds with fewer instances of complete dissociation with the target ASIP. This reflects that Ligand-1 (ZINC14539068) maintains closer and continuous contact through hydrogen bonds with protein ASIP, enhancing its inhibitory potential through stronger interactions. On the other hand, from the estimation of the hydrogen bond distance between Ligand-1 and the interacting residues of the ASIP, Ligand-1 showed four hydrogen bonding interactions during the simulation time with ASIP. The depicted hydrogen bond (HB) distance graph includes each c from the MD simulation trajectory. The amino acid residues CYS114, CYS116, and VAL127 interacted with Ligand-1 with average HB distances of 3.1 Å, 4.1 Å, and 3.3 Å (Figure 7C). The interaction of these residues with Ligand-1 showed less strong hydrogen bonding, based on the distance of the hydrogen bond interaction of Ligand-1 with ASIP. However, the ASIP SER129 residue that interacted with Ligand-1 showed an average hydrogen bond distance of 2.8 Å, conferring a strong interaction between Ligand-1 and ASIP. All four bonds showed bond distance fluctuation from 1.9 to 13 Å during the 100 ns simulation time period. However, the average distances of the hydrogen bonds established between Ligand-2 and ASIP amino acid residues ARG126, CYS116, VAL127, and SER129 were 3.2 Å, 4.9 Å and 5.3 Å, and 3.4 Å (Figure 7D). The interaction of Ligand-2 showed less strong hydrogen bonding, based on the established distance of the hydrogen bond interaction of Ligand-2 with ASIP. The hydrogen bond occupancy percentages between Ligand-1 and ASIP amino acid residues CYS114, CYS116, VAL127 and SER129 were 33%, 42%, 45% and 52%. However, the percent hydrogen bond occupancies between Ligand-2 and ASIP amino acid residues ARG126, CYS116, VAL127, and SER129 were 37%, 25%, 22% and 32%.

## 3. Discussion

In this study, we investigated a receptor-based indirect strategy by engaging the C-terminal groove of ASIP to modulate melanocortin signaling rather than MC1R. We were guided by previously reported structural and chimera data that stipulate the ASIP C-terminal loop is sufficient for inverse agonism of MC1R [24]. As reported, the ASIP structure is subdivided into three different loops. The N-terminal loop touches MC1R’s EC2/EC3 loops. The active loop (with the RFF motif) comes near the receptor mouth but should not trigger signaling. The LVARAA sequence of human MC1R and the VLSLN motif at the C-terminal of ASIP have been reported to engage in hydrophobic interactions [16]. Additionally, it is also known that after LVARAA and VLSLN interaction, the highly conserved RFF active domain (Arg-Phe-Phe) triplet of ASIP present in the C-terminal loop relocates exactly to the same binding pocket of MC1R as the HFRW (His–Phe–Arg–Trp, positions 6–9) motif of α-MSH, thereby activating the receptor in a similar fashion. This binding pocket domain is believed to partially overlap with the binding site of α-MSH and hence disrupts the proper melanocortin signaling [17,18,19].

This study is specifically focused on the C-terminal loop instead of taking the full ASIP sequence (PDB ID:2KZA). After structural superimposition of the ASIP–MC1R complex with the α-MSH–MC1R complex, we also found that both ASIP and α-MSH bind in the same pocket. This indicates that the ASIP mimics α-MSH and competes for the same binding pocket on MC1R, potentially affecting α-MSH binding and modulating receptor activity accordingly. The observed overlap between the ASIP and α-MSH binding pockets on MC1R provides strong structural support for a competitive antagonism mechanism via steric exclusion. However, the present docking and superimposition analyses do not directly assess receptor conformational states or downstream signaling outcomes. While ASIP has been experimentally described as an inverse agonist of MC1R in prior studies, the current work is limited to a structure-based hypothesis and does not attempt to functionally distinguish ligand displacement from stabilization of an inactive receptor conformation. Based on this mechanism, we tried to inhibit the ASIP binding to MC1R to allow α-MSH binding at its native MC1R receptor pocket, potentially to restore the normal signaling process. To explore this, ASIP was considered as a pharmacophoric template to find a small molecule against the VLSLN region of ASIP’s domain.

Three-dimensional pharmacophores encode both the type and the 3D arrangement of ligand features that drive recognition at a macromolecular binding site. It also describes what, where, and how the ligand interacts and specifies which interaction type matters the most. Individual chemical functionalities are abstracted into general feature classes—for example, hydrogen bond acceptors/donors (HYAs/HYDs), hydrophobes (HYs), aromatic rings, and positively or negatively ionizable centers. Beyond feature identification and positioning, we can also predict the directionality (e.g., the vector of an H-bond, π-interactions, or the orientation of interaction). Each feature carries a tolerance radius and weight, ensuring control over how strictly its location is tuned and how significantly it influences scoring (fit value). To capture the steric shape of the binding pocket and prevent unrealistic possession, pharmacophores are typically paired with excluded-volume spheres—geometric regions that mimic protein atoms and prevent poses that ligands could not recognize or occupy within the receptor [25]. The target ASIP-based pharmacophore model was prepared, which served as the central dogma to guide the screening of ligands against the ASIP C-terminal interface. Our structure-based pharmacophore captures key descriptors at the ASIP C-terminal interface with moderate sensitivity and high specificity. Using a dataset of 10 putative actives and 8 putative inactives/decoys, the generated pharmacophore model mapped 6 out of 10 putative actives, presenting 0.60 sensitivity, while rejecting all 8 out of 8 decoys, showing specificity as 1.00. The enrichment factor at six hits (≈1.8) shows that the model retrieves putative actives ~1.8× better than random selection. Mechanistically, the requirement that Hydrophobe63 be satisfied and that ≥1 feature associated with the VLSLN domain focuses the hypothesis on the MC1R-ECL1 site implicated in ASIP–MC1R interaction. All six mapped ligands, including Hydrophobe63, satisfied 4 out of 11 selected features and depicted significant interaction geometry within the VLSLN pocket. The fit value distribution also discriminated mapped ligands from unmapped ligands.

Based on these minimal features (HBA, HBA, HY/aromatic points, and excluded volumes), to focus on the ASIP’s EC1-facing contacts, we curated CHEMBL5173264 (with the highest fit value and completely mapped features) as an appropriate input for expected virtual screening in the ZINCPharmer database. The pharmacophore allowed us to align diverse groups of compounds based on the fit value, which indicates how well they map to the model features, retaining only those poses that significantly reproduce key ASIP interactions.

However, pharmacophore agreement alone does not fully ensure a viable lead candidate. We therefore coupled it with ADMET and toxicity and docking/ΔG triage to confirm shape complementarity to gauge developability from the outset. Compounds were further selected only if they fell into the pragmatic physicochemical criteria (e.g., MW, cLogP, TPSA, HBD/HBA, and rotatable bonds) that include predicted permeability and solubility under an acceptable range to avoid high-risk liabilities (PAINS alerts, significant hERG/CYP inhibition, Ames’ positivity score, and hepatotoxicity flags). This pharmacophore-based docking and ADME/Tox integrated funnel suppresses false positives, improves and prioritizes only those ligands that can maintain ASIP engagement, and increases the chance that the final hits will translate into stable, safe chemical matter suitable for further experimental downstream validation and lead optimization [18,26,27].

As per our observation in this study, the screened drug-like candidates based on fit value, binding affinity, and in silico ADMET/tox can be predicted to bind effectively with the VLSLN (EC1-facing surface) of ASIP. Due to ligand binding at this region, we predicted that the conformational changes in the ASIP structure might misorient the RFF active loop, lowering the chance that RFF engages the α-MSH/HFRW-like agonist pocket on MC1R [16]. Previous studies tested this interface mainly with mutations or peptide chimeras (for example, swapping loops or alanine scanning the RFF region) [28]. Our docking strategy aims to prevent the interaction of ASIP with MC1R by inhibiting the loop with ligand binding. This should lower ASIP’s ability to compete with α-MSH at MC1R and, in turn, help preserve eumelanogenic signaling. The selected ligands, Ligand-1 (ZINC14539068) and Ligand-2 (ZINC24890597), which showed the best binding affinity in docking studies, were further evaluated through 100 ns molecular dynamics simulations to assess the stability of their complexes with ASIP. The RMSD profiles indicated that both ligand–protein complexes remained dynamically stable throughout the simulation period. The Ligand-1–ASIP complex stabilized around an average RMSD of ~3 Å after initial deviations, comparable to the native ASIP system (~2.8 Å), suggesting that Ligand-1 binds stably while allowing moderate flexibility within the binding pocket. The Ligand-2–ASIP complex exhibited slightly higher RMSD values (~4 Å), reflecting increased local flexibility at the binding site without compromising overall structural integrity. These results highlight that the candidate ligands can form stable interactions with ASIP while accommodating necessary conformational adjustments. Understanding such dynamic behavior is critical for optimizing ligand efficacy and informing rational design strategies for potential ASIP-targeted therapeutics [29]. The RMSF analysis of ASIP indicates that the candidate compound (Ligand-1) modulates the conformational flexibility of critical domains in a manner analogous to the non-ligand-binding ASIP, supporting their potential binding stability and functional efficacy. The significance of the low RMSF values from the RMSF analysis is that it is an essential tool in the identification of the rigid and flexible sections of the protein structure [30]. Overall, the RMSF data confirms that ligand binding reduces the conformational mobility of ASIP, particularly within the active site, supporting the formation of a stable complex. The structural compactness and fluctuation stability directly affect ligand efficacy. The more stable ligand–ASIP complex suggests that this ligand may induce a favorable conformational state in ASIP, enhancing its inhibitory potential of the ligand. RG analysis also supported these findings, with the Ligand-1–ASIP complex exhibiting comparable and stable compactness as ASIP throughout the simulation period. RG analysis primarily supports the structural stability and consistency of the complexes rather than a biologically significant increase in compactness. A stable network of intermolecular interactions between the protein and ligand was preserved throughout, supporting the overall conformational stability of the complexes across the simulation timescale [31,32].

Post-MD simulation MM-GBSA binding free energy (dG) calculations further supported this observation, with Ligand-1 exhibiting the most favorable binding energy (−103 ± 4 kcal/mol). In comparison, Ligand-2 showed a less favorable binding free energy (−91 ± 3 kcal/mol), indicating weaker binding affinity. Together, the compact structural behavior and lower binding free energy confirm the superior stability and binding strength of Ligand-1 toward ASIP.

The hydrogen bond between protein–ligand complexes is one of the key non-covalent interactions to maintain stable molecular interaction recognition and complex stability [33]. The molecular dynamics simulation was also performed to analyze the interactions between the ligand and the residues of key amino acids of the binding pocket, such as ARG126, VAL127, SER129, and LEU130, which showed the establishment of hydrogen bonds within the stable complex formed by the compounds Ligand-1 and Ligand-2 with ASIP (Figure 7). Key amino acid residues of the binding pocket of the target protein play a role in the stabilization of the binding of the candidate compounds [34]. The stability of the ligand–protein complex was further assessed by studying the variation in distance between Ca atoms of the interacting residue of the amino acid and Ligand-1 and Ligand-2 during all the conformations of MD simulations. This strong hydrogen bonding network plays a crucial role in maintaining the structural integrity of the ligand–protein complex, significantly enhancing both the binding affinity of the candidate compound and the overall conformational stability of the complex [32,35]. Based on the simulation result, the number of hydrogen bond establishments and the average HB distance between the compound ZINC14539068 (Ligand-1) and ZINC24890597 (Ligand-2) were determined. The hydrogen bonding analysis indicates that Ligand-1 maintains stable interactions with ASIP, consistently forming two to three hydrogen bonds throughout the 100 ns simulation. Notably, SER129 exhibited a strong interaction with Ligand-1, with a short average hydrogen bond distance (2.8 Å) and the highest occupancy (52%), contributing significantly to complex stability. In contrast, interactions with CYS114, CYS116, and VAL127 were moderate, showing longer distances and lower occupancies. Ligand-2 displayed weaker hydrogen bonding, characterized by longer average bond distances (3.2–5.3 Å) and lower occupancies (22–37%). Overall, the higher hydrogen bond persistence and shorter distances observed for Ligand-1 suggest stronger binding stability and improved inhibitory potential compared to Ligand-2. More fluctuation in the hydrogen bond distance is attributed to the less effective binding of the ligand with residues of the active pocket of the protein [36]. The average distance between the compounds and the hydrogen bond contributing residues should be near or below 3 Å, which falls within the accepted range [37]. The findings from this study highlight the potential of a compound with ID ZINC14539068 as a promising inhibitor ligand of ASIP, supported by pharmacophore mapping, molecular docking, and molecular dynamics simulation results demonstrating stable hydrogen bonding interactions. While minor fluctuations in hydrogen bond distance suggest some variability in binding efficiency, the overall interaction profile supports the structural compatibility of Ligand-1 within the ASIP active site. Importantly, the integration of structure-based pharmacophore modeling with multi-level computational and preliminary experimental approaches provides a robust framework for evaluating compound efficacy. The therapeutic relevance of disrupting ASIP–MC1R binding might be of importance if, instead of utilizing MC1R agonists such as afamelanotide to activate MC1R signaling irrespective of endogenous regulatory context, it leads to non-physiological receptor stimulation [38]. In contrast, ASIP functions as an endogenous antagonist and inverse agonist of MC1R [16]. Therefore, disrupting ASIP_MC1R binding represents a complementary strategy that aims to relieve pathological suppression of MC1R rather than force receptor activation. This approach may allow restoration of physiological α-MSH-mediated signaling with improved regulatory balance and potentially reduced adverse effects. Accordingly, targeting the ASIP–MC1R interface offers a mechanistically distinct and biologically relevant alternative to direct MC1R agonism. However, in vitro findings remain preliminary, underscoring the need for further validation in diverse cellular and in vivo models. Future investigations should focus on optimizing ligand binding affinity and selectivity to advance translational potential. This work lays a foundational step toward the rational design and development of ASIP-targeted therapeutics.

## 4. Materials and Methods

### 4.1. Preparation and Docking of ASIP with MC1R

The NMR solution structure of agouti signaling protein (ASIP) (PDB ID: 2KZA) (https://www.wwpdb.org/pdb?id=pdb_00002kza, accessed on 2 September 2025) and the cryo-EM structure of α-MSH-bound melanocortin-1 receptor (PDB ID:7F4F) were retrieved from the RCSB Protein Data Bank. The structure of proteins was prepared by applying the CHARMm force field, using BIOVIA Discovery Studio (BIOVIA Discovery Studio v24.1.0.321712). During protein preparation, unnecessary chains, heteroatoms, ligands not relevant to the study, and water molecules were removed. Hydrogen atoms were added, and missing atoms or residues were filled using the prepare protein method. Specific binding site spheres were generated as per the study of Patel et al., 2010 [16], for both ASIP and MC1R using the binding site analysis tool in Discovery Studio. These spheres were centered on catalytically or functionally important residues with the following coordinates: ASIP—XYZ: (22.589950, 10.667464, −10.407046), radius: 8.456096 Å; MC1R—XYZ: (142.995903, 89.391304, 89.861906), radius: 10.310777 Å.

Protein–protein (ASIP-MC1R) interaction was performed using the ZDOCK method Biovia (BIOVIA Discovery Studio v24.1.0.321712) [39], with an Euler angular step size of 6° to predict the binding orientation between ASIP and MC1R. The RMSD cutoff and interface cutoff were set to 6.0 Å and 9.0 Å, respectively. This configuration generated 54,000 docking poses, ensuring exhaustive sampling and higher accuracy. A subset of top-ranked docking poses was then selected for refinement and rescored using the RDOCK module to determine and evaluate the binding interactions, stability, and interface quality. For structural comparison and alignment, the docked complex was superimposed onto the original ASIP structure (PDB ID: 2KZA) to validate and analyze consistency and potential changes in conformation upon binding.

### 4.2. Receptor-Based Pharmacophore Modeling

In this study, a structure-based pharmacophore (SBP) modeling approach of Biovia DS software (BIOVIA Discovery Studio v24.1.0.321712) was employed, which relies on information derived from the receptor’s active site to generate pharmacophore features. Specifically, the known ASIP–MC1R interaction interface was utilized, centered at the coordinates (22.523241, 10.701949, −10.362949) with a sphere radius of 8.032832 Å—corresponding to the ASIP binding region previously docked with MC1R [40].

A Ludi interaction map was generated using the Interaction Generation protocol available in BIOVIA Discovery Studio (BIOVIA Discovery Studio v24.1.0.321712). This map produces a large number of vectorized interaction features, including hydrogen bond acceptors (HBAs), hydrogen bond donors (HBDs), and hydrophobes (HYs), to comprehensively capture all potential ligand and protein interaction hotspots within the defined binding pocket. However, many of these generated features are redundant, spatially overlapping, or represent very weak interaction points; therefore, it is suggested by the Discovery Studio protocol that utilizing all of them is not a good idea for pharmacophore-based screening.

To construct a robust, reliable, and generalizable pharmacophore model, the LUDI-generated features were systematically clustered and filtered, and only non-redundant representative features orienting towards the ASIP–MC1R interaction interface were kept. The features that were located within the VLSLN binding region and pointing toward the core pocket were prioritized as interaction vectors, while the peripheral and overlapping features were excluded. This reduction resulted in a final set of 11 pharmacophoric features, which capture the dominant interaction requirements of the ASIP binding pocket while avoiding excessive model complexity. Using all LUDI-generated features would likely increase artificial enrichment and overfitting without improving predictive performance [26].

### 4.3. Pharmacophore Model Validation

Validation of the generated pharmacophore model is essential to ensure its reliability in distinguishing active-like ligands from putative inactive controls and in capturing meaningful geometric and chemical features relevant to true protein–ligand interactions [41]. This step also confirms the quality of the pharmacophore features and their suitability for downstream virtual screening. The general validation strategy was adapted from established pharmacophore-based screening workflows, including the study of Temml et al. (2017) [41], which emphasizes the use of ligand sets to assess model discrimination performance.

Because no experimentally validated ASIP ligands have been reported in the literature or public chemical repositories, a structure-based docking workflow was used to generate a surrogate dataset for internal pharmacophore validation. Approximately 598,260 diverse small molecules were retrieved from the ChEMBL database (https://www.ebi.ac.uk/chembl/, accessed on 10 September 2025) prepared, and docked into the predicted ASIP binding pocket using the LibDock scoring function (BIOVIA Discovery Studio v24.1.0.321712). Docking prioritized predicted binding affinity and the presence of key site-specific interactions in order to identify potential ASIP binding candidates. From this large-scale virtual screen, the top 10 ligands with the highest LibDock scores and favorable binding poses were selected as putative active-like compounds.

To evaluate model specificity, a set of 8 structurally diverse kinase inhibitors with no known or predicted interaction with ASIP was used as putative inactive controls. Together, these 18 ligands (10 putative actives + 8 putative inactives) formed the validation dataset used to assess pharmacophore performance (Table 1).

Pharmacophore validation was conducted using the Ligand Pharmacophore Mapping protocol in BIOVIA Discovery Studio. A key hydrophobic feature located within the ASIP pocket, Hydrophobe63, was assigned as a required feature to ensure that all mapped ligands engaged the central hydrophobic region. Additionally, a feature group representing critical hydrophobic interactions within the VLSLN C-terminal binding region was defined to further refine pocket selectivity. Screening parameters were set as follows: maximum features = 8, minimum features = 4, and maximum subset pharmacophores = 250. According to Discovery Studio’s mapping criteria, a ligand was required to match at least four pharmacophore features to be considered a valid hit.

This internally generated, docking-driven validation framework enabled assessment of the pharmacophore model’s ability to preferentially recognize predicted ASIP binders while excluding structurally unrelated non-binders, thereby establishing its suitability for subsequent large-scale virtual screening.

### 4.4. Virtual Screening Based on Pharmacophore

To perform virtual screening, the ZINCPharmer web tool (http://zincpharmer.csb.pitt.edu/pharmer.html, accessed on 2 September 2025) was utilized to screen the compounds from the ZINC purchasable database. ZINCPharmer is an online database that can search approximately 176 million conformations in just a few minutes by using the Pharmer pharmacophore search technology [42]. Based on the pharmacophore fit value, ligand CHEMBL5173264 with the highest fit value (3.25219) was selected, and its four pharmacophore-mapped features were defined as the query input Acc18 (hydrogen bond acceptor)xyz: (22.753500, 6.078500, −10.516000), Donor73 (hydrogen bond donor)xyz: (27.067500, 7.365000, −11.894500), Hydr63 (hydrophobic feature)xyz: (21.065000, 3.173000, −11.755000), Hydro72 (hydrophobic feature)xyz: (17.846000, 9.101000, −8.168000). The ZINCPharmer search was further refined to include only compounds with a molecular weight between 350 and 500 Da. This search yielded 4025 hit compounds, which were subsequently prepared, duplicates removed, and energy minimized to assess their potential as lead candidates for ASIP [43].

The curated ligands were further utilized for comprehensive ADMET profiling.

### 4.5. Pharmacokinetic and Toxicological Profiling

Pharmacokinetic and toxicity assessments were conducted using the ADMET Descriptors and TOPKAT toxicity prediction protocol of Discovery Studio Client (BIOVIA Discovery Studio v24.1.0.321712). A curated set of ligands, filtered for structural correctness and duplicates, underwent profiling for six primary ADMET properties: human intestinal absorption (HIA) via the AlogP98 vs. PSA_2D model (Levels 0 to 1 retained), aqueous solubility through a regression-based logS model (Levels 2 to 4 retained), blood–brain barrier (BBB) penetration classified by logBB values (Levels 1 to 3 retained), CYP2D6 inhibition via Bayesian classification (score less than 0.160 prioritized), hepatotoxicity predicted by structure–activity relationship models (non-toxic were retained), and plasma protein binding (PPB), with high binders (greater than or equal to 90%) deprioritized. Additionally, comprehensive toxicity screening was carried out using the TOPKAT module, which included four endpoints: weight-of-evidence (WOE) carcinogenicity prediction, AMES mutagenicity, skin irritancy, and skin sensitization. It employs a range of robust, cross-validated quantitative structure–toxicity relationship models for assessing various measures of toxicity. Ligands predicted as non-carcinogenic, non-mutagenic, and non-irritant or sensitizers were selected. Those compounds satisfying all the ADMET and TOPKAT toxicity filters were shortlisted and considered as drug-like candidates for further computational and in silico validation [44,45].

### 4.6. ASIP Docking with Drug-like Candidates

To evaluate the binding affinities and interaction profiles of 37 curated drug-like small molecules that passed prior ADMET and TOPKAT toxicity filters with ASIP, molecular docking was performed using the −CDOCKER module of BIOVIA Discovery Studio v24.1.0.321712. −CDOCKER is a CHARMm-based molecular docking tool that enables full ligand flexibility, accounting for variations in torsion angles, bond angles, and bond lengths, thereby generating highly accurate docked conformations.

Subsequently, another docking method, LibDock, was employed to further evaluate and rank the docked ligands using hotspot-guided placement and shape complementarity, allowing robust and efficient comparison of binding modes and relative affinities across the compound set. Additionally, AutoDock docking based on the Lamarckian genetic algorithm was performed independently to validate binding orientations and binding affinities. AutoDock predicted binding free energies and inhibition constants were used to cross-validate the ligand ranking score to ensure robustness and consistency of docking outcomes across multiple scoring functions [46,47]. Docking was focused on the ASIP–MC1R binding interface, specifically targeting the conserved VLSLN motif, which is critical for receptor recognition. The same binding coordinates used in pharmacophore modeling were retained to ensure methodological consistency. The docking sphere was defined with center coordinates (X = 22.589950, Y = 10.667464, Z = −10.407046) and a radius of 8.456096 Å. Each ligand was allowed to generate up to ten conformations (poses), and the −CDOCKER interaction energy score (−CDOCK Score) binding was computed for each pose. The pose exhibiting the most positive –CDOCK score was considered the most favorable and stable binding conformation. LibDock ranked poses with higher LibDock scores, and AutoDock poses with the lowest predicted binding free energies were considered optimal. Docked complexes were visualized in Discovery Studio, and interaction maps were generated to examine key interactions such as hydrogen bonds, hydrophobic contacts, π–π stacking, and van der Waals forces. Top ligands with strong docking scores (-CDOCK Score) were superimposed with pharmacophore features. Evaluating these features helps to design or optimize drug candidates with enhanced binding specificity and biological efficacy that are prioritized for post-docking evaluations. To describe the binding site in protein, the interaction binding affinity score (−CDOCK Score) and calculation of binding energy (ΔG) are likely internal steps within BIOVIA Discovery Studio v24.1.0.321712 [30].

### 4.7. Superimposition of Docked Ligands on Pharmacophore Features

To confirm the compatibility of the selected ligands with the pharmacophore features essential for ASIP binding, pharmacophore superimposition feature mapping was conducted for all five docked ligands, similar to the protocol employed earlier in this study. Pharmacophore characterization plays a pivotal role in molecular recognition by capturing essential features such as hydrogen bond acceptors (HBA), hydrogen bond donors (HBD), and hydrophobic regions, which are critical for receptor–ligand interactions. Evaluating these features helps to design or optimize drug candidates with enhanced binding specificity and biological efficacy.

### 4.8. Molecular Dynamic Simulation (MDS) and Binding Energy Calculation (MM-GBSA)

Molecular dynamics (MD) simulations were carried out using BIOVIA Discovery Studio v24.1.0.321712. Based on docking score, binding energy, and conformational pose analysis, the best complexes (ASIP–ligand) were selected for molecular dynamics (MD) simulation [35,48]. The best predicted top hits of ligands with respect to ASIP were selected to perform 100 nanoseconds (ns) through the standard dynamics cascade method. To generate the molecular topology files for the ASIP and ASIP–ligand complex and to create the topology of ligands, the Charmm36 force field was used. The solvation system consists of an explicit boundary TIP 3-point water solvent model, an orthorhombic box with a minimal distance of 7 nm between the protein surface and the edge of the box neutralized with the inclusion of cation-type sodium (Na) and anion-type chloride (Cl) counter ions [49]. After solvation of the protein and complex, the standard dynamics cascade method was employed. For energy minimization, the steepest descent (minimization 1) for 1000 steps with RMS gradient 1 and conjugate gradient (minimization 2) for 4000 steps with RMS gradient 0.1. Both minimization algorithms were used for a total of 5000 steps. The heating phase was performed using a simulation time of 50 picoseconds (ps) with a time step of 2 fs; for immersion, the initial temperature is 50, and the target temperature is 300 K with a save results interval of 10 ps. The reference temperature was 300 K, and the reference pressure was 1.0 bar for the NPT (isothermal–isobaric) ensemble. The equilibration phase was carried out for a 200 ps simulation run with a 2 fs (femtosecond) time step, and the save result interval is 10 ps to generate the restart file for further equilibration of the complex. The Particle_Mesh_Ewald (PME) algorithm was used for long-range electrostatic interactions with fourth-order cubic interpolation and a kappa 0.34 Å grid spacing. The advanced dynamic integrator used the Leapfrog Verlet algorithm with the applied shake constraint. The explicit solvent model was used with a dielectric constant of 1, a nonbond list radius cutoff of 14 Å, in which the nonbond higher cutoff distance is 12 Å and the nonbond lower cutoff distance is 10 Å. The production step of a standard dynamic cascade of MD simulation was carried out for 100 ps with a save result interval step of 10 ps. Following the complete run of the standard dynamics cascade, the output restart file from the standard dynamics cascade was used to run the dynamics equilibration protocol to equilibrate the complex system. After equilibration of the complex for 5000 ps, followed by the dynamics production step to complete the 100 ns molecular dynamics simulation for ASIP and ASIP–ligand complex. The analysis trajectory method was used to confirm the established interaction between ligand and protein in each conformation or frame of the simulation trajectory. Several key parameters were analyzed to understand the dynamics of the system. The root mean square deviation (RMSD), root mean square fluctuation (RMSF), and radius of gyration (RG) were calculated to measure the overall stability of the protein in the absence and presence of ligand by tracking how much the atoms moved relative to a reference structure [50,51]. The stability of the complex in each frame was further confirmed by monitoring established hydrogen bonds between protein and ligand interacting atoms. The stability of the complex is indicated by the highest potential inhibitor from the stable complex protein–ligand through BIOVIA Discovery Studio v24.1.0.321712. Additionally, the binding free energy of conformations between the receptor and the best hits was calculated by molecular mechanics with generalized Born surface area (MM-GBSA) using BIOVIA Discovery Studio v24.1.0.321712 as described previously [30]. The MM-GBSA (dG) was calculated for both pre-MD Simulation (best docking hits) and post-MD Simulation for selected ligand–protein complexes from simulation confirmations.

## 5. Limitations

This study presents a receptor-based pharmacophore model for ASIP; however, methodological limitations must be acknowledged. ASIP is a largely unexplored target for small molecule modulation, and, to date, no experimentally validated ASIP inhibitors have been reported in the literature or public databases. Hence, the validation strategy relied on docking-enriched putative actives and chemically unrelated putative inactive controls, rather than true experimentally confirmed binders and non-binders. As such, the pharmacophore validation presented here should be regarded as an internal, docking-based consistency check, conceptually analogous to actives–decoys benchmarking, rather than a full experimental validation. Consequently, the performance metrics (sensitivity, specificity, and enrichment factor) reflect the model’s discrimination ability within a surrogate dataset and may not fully capture behavior against real ASIP ligands. Future work involving biochemical binding assays, experimentally curated actives, or prospectively validated screening hits will be required to refine and externally validate the pharmacophore model.

## 6. Conclusions

Our study suggests ZINC14539068 as a new potent inhibitor of ASIP to preserve α-MSH-mediated signaling of MC1R. This finding will help reduce pheomelanin-associated oxidative stress. Using an integrated triage pipeline (structure-based pharmacophore/docking/ADME–tox), we identified compounds that are predicted to bind with the ASIP C-terminal hydrophobic groove, which disrupts its antagonistic competent geometry, discouraging ASIP’s RFF engagement to the MC1R agonist site. Short-term molecular dynamics simulations supported the stability of ZINC14539068 at the targeted pocket and the persistence of key contacts, reinforcing its prioritization and validating the docking and pharmacophore-based rankings. To the best of our knowledge, this is the first study that purposefully targets ASIP itself, rather than modifying or targeting MC1R to prevent ASIP from adopting the antagonistic competent conformation. By weakening ASIP’s C-terminal ability to compete at MC1R, our strategy is expected to favor the physiological α-MSH agonism and cAMP signaling, offering control of the melanocortin and shifting melanogenesis from pheomelanin to eumelanin. Eumelanin provides superior UV shielding, more radical scavenging, and reduces DNA damage in skin, thereby being associated with a lower risk of melanoma and other skin diseases. Our study also provides tractable starting points for medicinal chemistry and motivates experimental validation in melanocytic models, followed by in vivo evaluation.

## Figures and Tables

**Table 1:** Putative active ligands selected for pharmacophore validation, showing LibDock score, pharmacophore fit value, mapped pharmacophore features, and the 2D structure of ligands.

S. No.	Putative Active Ligand (Compound ID)	LibDock Score	Pharmacophore Fit Value	Mapped Pharmacophore Features	Structure
1	CHEMBL 5173264	55.67	3.25219	Acceptor18, Donor73, Hydrophobe63, Hydrophobe72	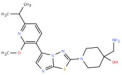
2	CHEMBL 3704007	23.22	2.35146	Acceptor18, Donor73, Hydrophobe63, Hydrophobe72	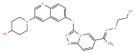
3	CHEMBL 1160704	61.69	1.26965	Acceptor9, Hydrophobe63, Donor73, Donor123	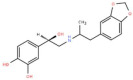
4	CHEMBL 1396943	59.06	1.14727	Acceptor18, Hydrophobe63, Hydrophobe72, Donor73	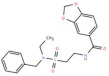
5	CHEMBL 388214	60.94	2.91306 × 10^−6^	Acceptor9, Acceptor10, Donor79, Donor125, Hydrophobe63	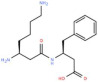
6	CHEMBL 4637051	43.26	0.00594556	Acceptor18, Donor73, Hydrophobe63, Hydrophobe72	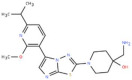
7	CHEMBL 4870623	77.53	-	-	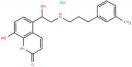
8	CHEMBL 3402193	61.20	-	-	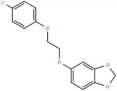
9	CHEMBL 3706834	27.76	-	-	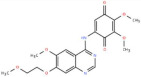
10	CHEMBL 202342	52.89	-	-	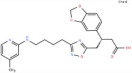

LibDock score represents docking pose quality based on receptor–ligand interaction geometry. Fit value reflects the geometric alignment of the ligand to the pharmacophore model.

**Table 2:** TOPKAT-predicted toxicity test for selected virtual screening hits, including carcinogenicity, mutagenicity, skin irritancy, and skin sensitization.

S. No.	Compound ID	WOE Carcinogenicity Prediction	WOE Score	AMES Prediction	AMES Score	Skin Irritancy	Skin Sensitization
1	ZINC71745364	NC	−3.994	NM	−7.12	None	None
2	ZINC56156219	NC	−3.361	NM	−7.79	None	None
3	ZINC71124054	NC	−2.280	NM	−8.013	None	None
4	ZINC16135691	NC	−1.018	NM	−19.63	None	None
5	ZINC24890597	NC	−5.572	NM	−10.39	None	None
6	ZINC91321775	NC	−6.562	NM	−18.59	None	None
7	ZINC90689169	NC	−2.910	NM	−16.64	None	None
8	ZINC19591955	NC	−4.477	NM	−16.12	None	None
9	ZINC05577112	NC	−3.908	NM	−6.54	None	None
10	ZINC92760749	NC	−5.400	NM	−20.03	None	None
11	ZINC64074154	NC	−2.438	NM	−10.33	None	None
12	ZINC14539068	NC	−6.713	NM	−8.78	None	None
13	ZINC36616718	NC	−3.792	NM	−7.87	None	None
14	ZINC20150420	NC	−3.355	NM	−12.62	None	None
15	ZINC71938586	NC	−7.300	NM	−20.21	None	None
16	ZINC14740395	NC	−7.205	NM	−9.97	None	None
17	ZINC59086944	NC	−3.034	NM	−9.090	None	None
18	ZINC57438376	NC	−3.646	NM	−6.214	None	None
19	ZINC64926416	NC	−0.963	NM	−5.098	None	None
20	ZINC19710117	NC	−4.349	NM	−6.162	None	None
21	ZINC19285359	NC	−2.585	NM	−13.00	None	None
22	ZINC15969989	NC	−5.645	NM	−12.04	None	None
23	ZINC15277589	NC	−4.574	NM	−10.96	None	None
24	ZINC63376144	NC	−1.836	NM	−12.02	None	None
25	ZINC20757631	NC	−5.685	NM	−9.595	None	None
26	ZINC12247721	NC	−3.248	NM	−11.14	None	None
27	ZINC12212035	NC	−1.454	NM	−7.043	None	None
28	ZINC57607890	NC	−3.956	NM	−14.89	None	None
29	ZINC21019835	NC	−4.820	NM	−10.57	None	None
30	ZINC24890743	NC	−11.03	NM	−20.06	None	None
31	ZINC20757502	NC	−5.195	NM	−9.495	None	None
32	ZINC20760260	NC	−5.987	NM	−12.52	None	None
33	ZINC00927842	NC	−4.429	NM	−11.69	None	Strong
34	ZINC15303567	NC	−5.213	NM	−8.92	None	None
35	ZINC57608004	NC	−4.572	NM	−16.19	None	None
36	ZINC20756673	NC	−5.497	NM	−12.5	None	None
37	ZINC63383785	NC	−1.205	NM	−9.47	None	None

NC: non-carcinogenic; NM: non-mutagenic. WOE and AMES scores are outputs from the TOPKAT predictive models.

**Table 3:** Docking scores, pharmacophore fit values, and binding free energies of prioritized ASIP ligands.

Best Ligand	Fit Value	−CDOCK Score	Libdock_ Score	AutoDock Binding Affinity (ΔG) (kcal/mol)	Pre-MDS MM-GBSA (dG) (kcal/mol)
ZINC14539068	1.9	35.38	37	−5.8	−65.88
ZINC24890597	1.7	28.31	38	−5.4	−63.38
ZINC12212035	1.4	25.29	33	−5.2	−60.59
ZINC64926416	1.3	33.22	33	−5.1	−25.03
ZINC91321775	0.038	29.26	35	−5.2	−15.61

Fit value, pharmacophore mapping fitness score; −CDOCK score, negative CDOCKER interaction energy—higher positive values indicate stronger predicted binding; LibDock score, hotspot-guided docking score; AutoDock binding affinity (ΔG), predicted binding free energy (kcal/mol); pre-MDS MM-GBSA (dG), free energy estimated prior to molecular dynamic simulation (kcal/mol).

## Data Availability

The raw data supporting the conclusions of this article will be made available by the authors upon request.

## Supplementary Materials

The following supporting information can be downloaded at https://www.mdpi.com/article/10.3390/ph19010114/s1, Figure S1. Ramachandran plot of the ASIP (PDB ID: 2KZA) shows that the majority of residues occupy favored and additionally allowed regions. Figure S2. The docked confirmations shown with -CDOCK score, binding energy and Fit Value score. The interaction of ASIP protein with ligands (A) ZINC12212035 (B) ZINC14539068 (C) ZINC24890597 (D) ZINC64926414 and with (E) ZINC1321775 to the binding pocket residues. Figure S3. Two-dimensional (2D) interaction diagram of the top-ranked ligand (A) ZINC14539068 (Ligand-1) and (B) ZINC24890597 (Ligand-2) docked into the ASIP binding pocket, highlighting hydrogen bonding, hydrophobic, alkyl, and π–alkyl interactions with key residues involved in MC1R recognition. Table S1. Predicted ADMET properties level of selected compounds. Table S2. Predicted values of ADMET property of selected compounds.

## Abbreviations

ASIP	Agouti signaling protein
MC1R	Melanocortin-1 receptor
α-MSH	α-melanocyte-stimulating hormone
ROS	Reactive oxygen species
RMSD	Root mean square deviation
RMSF	Root mean square Fluctuation
RG	Radius of gyration
