# Supplementary material for: Blocking ASIP to Protect MC1R Signaling and Mitigate Melanoma Risk: An In Silico Study
Source: Pharmaceuticals (Basel). 2026 Jan 8;19(1):114. doi: 10.3390/ph19010114 (PMC12844864; doi:10.3390/ph19010114)
Supplement: Supplementary file 1 [file pharmaceuticals-19-00114-s001.zip › pharmaceuticals-4037153-supplementary.pdf]

Supporting Information/Supplementary Material

Blocking ASIP to Protect MC1R Signaling to Mitigate Melanoma Risk: An in-silico study

Farah Maarfi [1], Mohammed Cherkaoui [1], Sana Afreen[2] and Mohd Yasir Khan [1,*]

1 Department of Digital Engineering and Artificial Intelligence, College of Science, Long Island University, Brooklyn-11201, New York, USA.
2 Department of Food and Nutrition, Era University, Lucknow-226003, Uttar Pradesh, India
* Correspondence: mohd.yasirkhan@liu.edu; Mohd Yasir Khan

Table of Contents

Figure S1. Ramachandran plot of the ASIP (PDB ID: 2KZA) shows that the majority of residues occupy favored and additionally allowed regions.

Figure S2. The docked confirmations shown with -CDOCK score, binding energy and Fit Value score. The interaction of ASIP protein with ligands **(A)** ZINC12212035 **(B)** ZINC14539068 **(C)** ZINC24890597 **(D)** ZINC64926414 and with **(E)** ZINC1321775 to the binding pocket residues.

Figure S3. Two-dimensional (2D) interaction diagram of the top-ranked ligand (A) ZINC14539068 (Ligand-1) and (B) ZINC24890597 (Ligand-2) docked into the ASIP binding pocket, highlighting hydrogen bonding, hydrophobic, alkyl, and π–alkyl interactions with key residues involved in MC1R recognition.

Table S1. Predicted ADMET properties level of selected compounds.
Table S2. Predicted values of ADMET property of selected compounds.

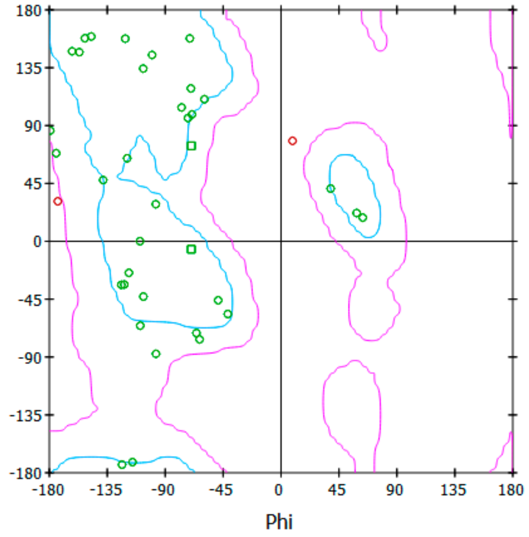

Figure S1. Ramachandran plot of the ASIP (PDB ID: 2KZA) shows that the majority of residues occupy favored and additionally allowed regions.

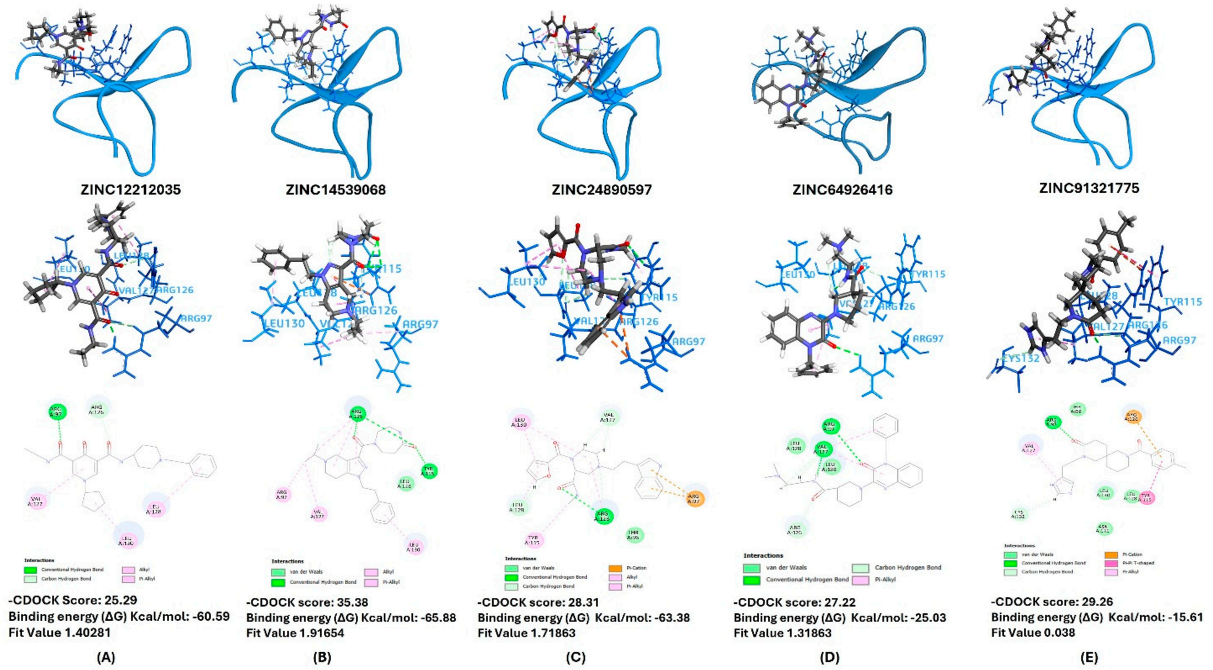

Figure S2. The docked confirmations shown with -CDOCK score, binding energy and Fit Value score. The interaction of ASIP protein with ligands **(A)** ZINC12212035 **(B)** ZINC14539068 **(C)** ZINC24890597 **(D)** ZINC64926414 and with **(E)** ZINC1321775 to the binding pocket residues.

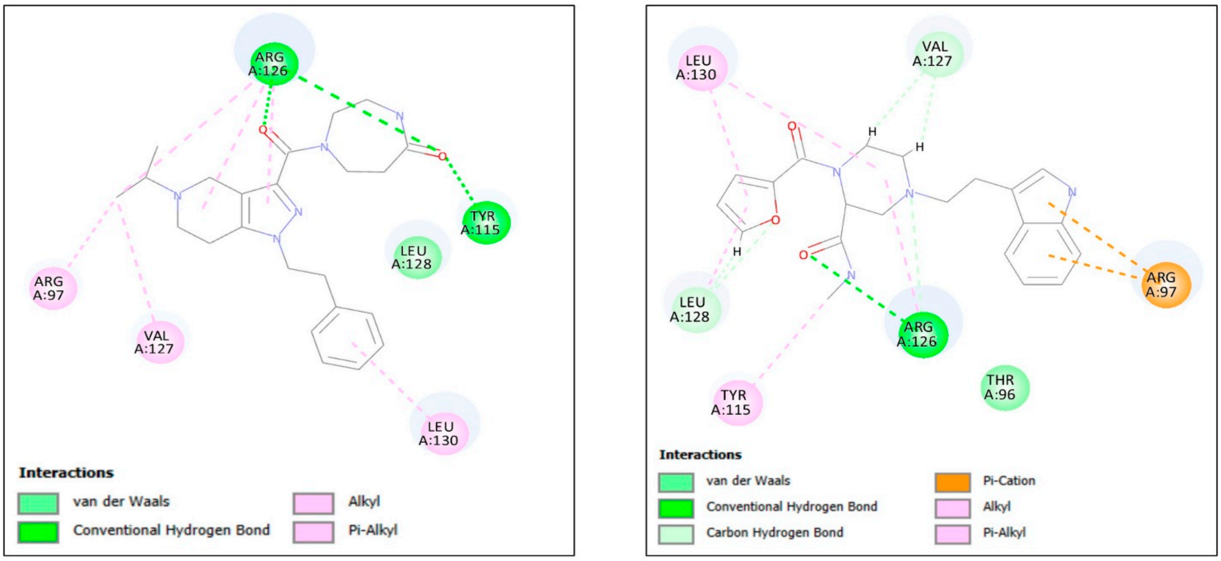

(A) **(B)**

Figure S3. Two-dimensional (2D) interaction diagram of the top-ranked ligand (A) ZINC14539068 (Ligand-1) and (B) ZINC24890597 (Ligand-2) docked into the ASIP binding pocket, highlighting hydrogen bonding, hydrophobic, alkyl, and π–alkyl interactions with key residues involved in MC1R recognition.

Table S1. Predicted ADMET classification levels for selected compounds generated using BIOVIA Discovery Studio.

S. No	Compound IDs	HI Absorption Level	AlogP98	PSA_2D	BBB Level	Solubility Level	CYP2D6	PPB Level	Hepatotoxicity Prediction
1	ZINC92674938	0	2.92	51.323	2	2	false	false	false
2	ZINC67654186	0	0.992	51.346	3	3	false	false	false
3	ZINC77162703	0	2.413	62.087	2	3	false	false	false
4	ZINC86329977	0	1.954	65.215	3	3	false	false	false
5	ZINC03847371	0	3.662	80.144	2	2	false	false	false
6	ZINC92869995	0	1.491	95.151	3	3	false	false	false
7	ZINC79967590	0	1.915	42.245	2	3	false	false	false
8	ZINC74102649	0	0.077	84.079	3	4	false	false	false
9	ZINC39486007	0	0.623	38.157	3	3	false	false	false
10	ZINC71745364	0	0.307	59.189	3	3	false	false	false

11	ZINC75483597	0	1.893	74.749	3	3	false	false	false
12	ZINC56156185	0	1.314	63.574	3	3	false	false	false
13	ZINC56156219	0	1.372	63.574	3	3	false	false	false
14	ZINC71124054	0	1.372	63.574	3	3	false	false	false
15	ZINC27666283	0	1.064	81.129	3	3	false	false	false
16	ZINC89815070	0	1.978	44.659	2	3	false	false	false
17	ZINC58159615	0	1.561	77.155	3	3	false	false	false
18	ZINC16135691	0	1.1	94.295	3	3	false	false	false
19	ZINC08132968	0	3.257	77.304	2	2	false	false	false
20	ZINC51131725	0	2.324	86.234	3	3	false	false	false
21	ZINC36170229	0	0.959	66.378	3	3	false	false	false
22	ZINC56156161	0	1.455	63.574	3	3	false	false	false
23	ZINC80718665	0	1.055	36.667	2	3	false	false	false
24	ZINC56162126	0	1.633	63.574	3	3	false	false	false
25	ZINC63411562	0	1.019	90.333	3	3	false	false	false
26	ZINC24890597	0	1.989	81.726	3	3	false	false	false
27	ZINC57466878	0	2.619	86.234	3	2	false	false	false
28	ZINC91321775	0	1.756	71.416	3	3	false	false	false
29	ZINC90689169	0	-0.278	74.621	3	4	false	false	false
30	ZINC19591955	0	1.652	38.513	2	3	false	false	false
31	ZINC56162139	0	0.634	50.615	3	4	false	false	false
32	ZINC46079144	0	2.057	92.198	3	3	false	false	false
33	ZINC05577112	0	2.53	95.565	3	2	false	false	false
34	ZINC92760749	0	0.514	83.617	3	4	false	false	false
35	ZINC71753791	0	1.959	61.268	2	3	false	false	false
36	ZINC24177950	0	1.829	51.722	2	3	false	false	false
37	ZINC64074154	0	1.287	49.477	3	3	false	false	false
38	ZINC14539068	0	2.233	70.726	3	3	false	false	false
39	ZINC63494561	0	2.595	78.664	3	2	false	false	false
40	ZINC36616718	0	3.019	51.876	2	2	false	false	false
41	ZINC35375520	0	0.344	76.07	3	3	false	false	false
42	ZINC20150420	0	1.866	85	3	3	false	false	false
43	ZINC71938586	0	2.653	63.425	2	3	false	false	false
44	ZINC14740395	0	2.757	70.726	2	2	false	false	false
45	ZINC20150424	0	1.585	85	3	3	false	false	false
46	ZINC59086944	0	0.356	70.309	3	3	false	false	false
47	ZINC57589692	0	0.799	93.261	3	3	false	false	false
48	ZINC57589692	0	0.799	93.261	3	3	false	false	false
49	ZINC57589445	0	0.799	93.261	3	3	false	false	false
50	ZINC57589621	0	0.799	93.261	3	3	false	false	false
51	ZINC57438376	0	0.539	89.656	3	3	false	false	false
52	ZINC64926416	0	0.7	68.644	3	3	false	false	false

53	ZINC35505628	0	1.127	85.882	3	3	false	false	false
54	ZINC19710117	0	-0.398	80.432	3	4	false	false	false
55	ZINC15000267	0	1.456	84.227	3	3	false	false	false
56	ZINC14986717	0	1.511	84.227	3	3	false	false	false
57	ZINC19285359	0	0.304	76.238	3	3	false	false	false
58	ZINC15970187	0	0.88	70.309	3	3	false	false	false
59	ZINC20566956	0	0.561	70.309	3	3	false	false	false
60	ZINC15969989	0	0.958	70.309	3	3	false	false	false
61	ZINC15277589	0	1.598	73.662	3	2	false	false	false
62	ZINC63514575	0	0.45	71.417	3	3	false	false	false
63	ZINC63478630	0	0.449	80.875	3	3	false	false	false
64	ZINC63383522	0	-0.268	68.065	3	3	false	false	false
65	ZINC63376144	0	-0.19	68.065	3	3	false	false	false
66	ZINC20757631	0	0.535	92.05	3	3	false	false	false
67	ZINC57589659	0	1.285	93.261	3	3	false	false	false
68	ZINC12247721	0	1.842	73.22	3	3	false	false	false
69	ZINC12212035	0	1.667	84.227	3	3	false	false	false
70	ZINC57607890	0	1.362	90.306	3	3	false	false	false
71	ZINC21019835	0	1.079	72.356	3	3	false	false	false
72	ZINC21880790	0	1.977	37.927	2	3	false	false	false
73	ZINC35375426	0	2.045	88.88	3	2	false	false	false
74	ZINC16025057	0	1.047	70.309	3	2	false	false	false
75	ZINC16025057	0	1.047	70.309	3	2	false	false	false
76	ZINC15969995	0	1.366	70.309	3	2	false	false	false
77	ZINC63383863	0	0.218	68.065	3	3	false	false	false
78	ZINC63376149	0	0.218	68.065	3	3	false	false	false
79	ZINC63478648	0	-0.101	68.065	3	3	false	false	false
80	ZINC24890743	0	1.976	69.023	3	2	false	false	false
81	ZINC20757502	0	-0.015	79.24	3	3	false	false	false
82	ZINC20760260	0	0.597	92.05	3	3	false	false	false
83	ZINC00927842	0	2.587	81.376	3	2	false	false	false
84	ZINC14990781	0	1.786	84.227	3	3	false	false	false
85	ZINC13511653	0	4.167	72.594	2	2	false	false	false
86	ZINC15303567	0	2.532	83.12	3	2	false	false	false
87	ZINC57608004	0	1.711	90.306	3	3	false	false	false
88	ZINC20756673	0	0.047	79.24	3	3	false	false	false
89	ZINC00928077	0	2.935	81.376	3	2	false	false	false
90	ZINC63383785	0	0.359	89.805	3	3	false	false	false
91	ZINC14540581	0	2.953	83.28	3	2	false	false	false
92	ZINC14732374	0	2.603	70.726	2	2	false	false	false
93	ZINC59286680	0	0.967	87.031	3	3	false	false	false
94	ZINC35804989	0	2.516	92.05	3	2	false	false	false

95	ZINC16026352	0	2.516	92.05	3	2	false	false	false
96	ZINC12571944	0	0.328	70.741	3	3	false	false	false
97	ZINC35375166	0	1.093	76.07	3	3	false	false	false

HI: Human Intestinal; BBB: blood brain barrier; PPB: plasma protein binding

Table S2. Numerical ADMET descriptor values for selected compounds predicted using BIOVIA Discovery Studio.

S. No	Compound IDs	HI Absorption Level	AlogP98	PSA_2D	BBB	Solubility	CYP2D6	PPB	Hepatotoxicity
1	ZINC92674938	0	2.92	51.323	-0.06	-4.557	-1.4411	-6.729	-7.81559
2	ZINC67654186	0	0.992	51.346	-0.66	-2.701	-0.6531	-5.351	-4.85061
3	ZINC77162703	0	2.413	62.087	-0.39	-3.572	-1.6878	-2.401	-5.01664
4	ZINC86329977	0	1.954	65.215	-0.582	-2.904	-1.9954	-4.532	-5.15671
5	ZINC03847371	0	3.662	80.144	-0.29	-4.063	-7.9553	-5.404	-4.89474
6	ZINC92869995	0	1.491	95.151	-1.19	-3.578	-6.2037	-2.717	-6.21172
7	ZINC79967590	0	1.915	42.245	-0.23	-3.254	-2.0430	-6.767	-9.94321
8	ZINC74102649	0	0.077	84.079	-1.46	-1.613	-4.8088	-6.459	-5.15875
9	ZINC39486007	0	0.623	38.157	-0.56	-2.532	-0.2480	-8.067	-8.65069
10	ZINC71745364	0	0.307	59.189	-0.99	-2.003	-6.8069	-5.102	-4.15846
11	ZINC75483597	0	1.893	74.749	-0.75	-3.482	-6.0427	-6.845	-6.81494
12	ZINC56156185	0	1.314	63.574	-0.75	-2.067	-2.3732	-9.043	-5.47222
13	ZINC56156219	0	1.372	63.574	-0.73	-2.127	0.0980	-11.06	-7.30869
14	ZINC71124054	0	1.372	63.574	-0.73	-2.108	-0.1062	-8.740	-7.1499
15	ZINC27666283	0	1.064	81.129	-1.10	-2.422	-6.4213	-2.786	-4.1694
16	ZINC89815070	0	1.978	44.659	-0.24	-3.477	-2.8872	-3.451	-6.71679
17	ZINC58159615	0	1.561	77.155	-0.89	-2.677	-7.4775	-2.589	-4.28209
18	ZINC16135691	0	1.1	94.295	-1.30	-2.444	-4.7561	-4.875	-4.1635
19	ZINC08132968	0	3.257	77.304	-0.37	-4.482	-3.1318	-3.179	-7.2528
20	ZINC51131725	0	2.324	86.234	-0.8	-3.931	-6.6103	-3.469	-7.42851
21	ZINC36170229	0	0.959	66.378	-0.90	-2.295	-2.9528	-5.040	-4.2065
22	ZINC56156161	0	1.455	63.574	-0.71	-2.349	-0.4705	-6.362	-4.761
23	ZINC80718665	0	1.055	36.667	-0.40	-2.708	-2.4966	-16.80	-7.18261
24	ZINC56162126	0	1.633	63.574	-0.65	-2.384	-2.7611	-9.521	-5.40431
25	ZINC63411562	0	1.019	90.333	-1.26	-2.104	-7.6173	-3.380	-6.82496
26	ZINC24890597	0	1.989	81.726	-0.83	-3.761	-9.2298	-21.27	-4.47734
27	ZINC57466878	0	2.619	86.234	-0.70	-4.065	-4.3359	-2.743	-4.97535
28	ZINC91321775	0	1.756	71.416	-0.74	-3.165	-6.7079	-8.453	-4.48067
29	ZINC90689169	0	-0.278	74.621	-1.42	-1.314	-5.7028	-13.45	-4.93764
30	ZINC19591955	0	1.652	38.513	-0.25	-3.432	-3.9906	-3.746	-6.18466
31	ZINC56162139	0	0.634	50.615	-0.75	-1.664	-2.6626	-7.656	-4.16252
32	ZINC46079144	0	2.057	92.198	-0.97	-3.31	-5.1957	-2.341	-4.60574

33	ZINC05577112	0	2.53	95.565	-0.88	-4.033	-0.5313	-3.397	-6.63306
34	ZINC92760749	0	0.514	83.617	-1.31	-1.454	-8.3255	-5.830	-6.28843
35	ZINC71753791	0	1.959	61.268	-0.51	-3.538	-7.1456	-6.493	-6.20577
36	ZINC24177950	0	1.829	51.722	-0.40	-3.114	-2.8071	-9.845	-5.34295
37	ZINC64074154	0	1.287	49.477	-0.53	-2.595	-3.1228	-10.41	-10.0581
38	ZINC14539068	0	2.233	70.726	-0.58	-3.728	-5.8143	-4.078	-8.8542
39	ZINC63494561	0	2.595	78.664	-0.59	-4.385	-3.6940	-3.038	-4.28352
40	ZINC36616718	0	3.019	51.876	-0.04	-4.766	-0.9468	-2.472	-4.56624
41	ZINC35375520	0	0.344	76.07	-1.25	-2.717	-6.5546	-8.135	-7.12731
42	ZINC20150420	0	1.866	85	-0.92	-3.707	-6.0409	-8.427	-10.8774
43	ZINC71938586	0	2.653	63.425	-0.33	-2.736	-7.7912	-12.05	-6.35225
44	ZINC14740395	0	2.757	70.726	-0.42	-4.065	-4.1279	-4.551	-8.89136
45	ZINC20150424	0	1.585	85	-1.00	-3.717	-4.9830	-7.211	-7.91106
46	ZINC59086944	0	0.356	70.309	-1.15	-3.382	-6.2214	-10.96	-4.87681
47	ZINC57589692	0	0.799	93.261	-1.38	-2.733	-10.725	-5.599	-4.91894
48	ZINC57589692	0	0.799	93.261	-1.38	-2.733	-10.725	-5.599	-4.91894
49	ZINC57589445	0	0.799	93.261	-1.38	-2.731	-10.658	-4.119	-6.18201
50	ZINC57589621	0	0.799	93.261	-1.38	-2.721	-10.053	-3.784	-6.62413
51	ZINC57438376	0	0.539	89.656	-1.40	-2.439	-10.415	-3.012	-4.82828
52	ZINC64926416	0	0.7	68.644	-1.02	-2.052	-7.2742	-4.844	-7.95914
53	ZINC35505628	0	1.127	85.882	-1.16	-2.816	-6.7256	-8.348	-5.2408
54	ZINC19710117	0	-0.398	80.432	-1.55	-1.05	-5.7137	-7.506	-4.36488
55	ZINC15000267	0	1.456	84.227	-1.03	-2.434	-3.3862	-4.585	-8.91517
56	ZINC14986717	0	1.511	84.227	-1.02	-2.768	-5.1701	-4.254	-7.74041
57	ZINC19285359	0	0.304	76.238	-1.26	-2.479	-3.7117	-6.221	-5.28947
58	ZINC15970187	0	0.88	70.309	-0.99	-3.723	-6.1747	-10.55	-5.23281
59	ZINC20566956	0	0.561	70.309	-1.09	-3.644	-7.4352	-10.26	-4.92852
60	ZINC15969989	0	0.958	70.309	-0.97	-3.625	-6.2946	-11.66	-5.59784
61	ZINC15277589	0	1.598	73.662	-0.82	-4.446	-4.5903	-9.674	-4.75056
62	ZINC63514575	0	0.45	71.417	-1.14	-3.176	-6.1429	-3.078	-6.02326
63	ZINC63478630	0	0.449	80.875	-1.29	-3.247	-8.5824	-3.752	-6.20122
64	ZINC63383522	0	-0.268	68.065	-1.31	-2.452	-7.7273	-3.646	-6.37198
65	ZINC63376144	0	-0.19	68.065	-1.29	-2.352	-7.8473	-4.757	-6.73701
66	ZINC20757631	0	0.535	92.05	-1.44	-3.556	-7.3347	-11.73	-5.20322
67	ZINC57589659	0	1.285	93.261	-1.23	-3.168	-9.3465	-4.771	-6.41159
68	ZINC12247721	0	1.842	73.22	-0.74	-3.306	-2.9555	-6.318	-9.26481
69	ZINC12212035	0	1.667	84.227	-0.97	-2.827	-2.9123	-5.830	-9.50656
70	ZINC57607890	0	1.362	90.306	-1.16	-3.261	-9.1449	-3.521	-6.13211
71	ZINC21019835	0	1.079	72.356	-0.96	-2.247	-10.905	-7.888	-4.87179
72	ZINC21880790	0	1.977	37.927	-0.14	-3.364	-2.4286	-4.451	-9.31634
73	ZINC35375426	0	2.045	88.88	-0.92	-4.458	-5.6602	-3.898	-4.77046
74	ZINC16025057	0	1.047	70.309	-0.94	-4.093	-8.1406	-8.901	-5.34092

75	ZINC16025057	0	1.047	70.309	-0.94	-4.093	-8.1406	-8.901	-5.34092
76	ZINC15969995	0	1.366	70.309	-0.84	-4.172	-6.8801	-9.186	-5.51168
77	ZINC63383863	0	0.218	68.065	-1.16	-2.903	-8.2450	-4.042	-4.97683
78	ZINC63376149	0	0.218	68.065	-1.16	-2.901	-9.7025	-2.647	-6.4936
79	ZINC63478648	0	-0.101	68.065	-1.26	-2.823	-10.557	-2.362	-6.09969
80	ZINC24890743	0	1.976	69.023	-0.63	-4.118	-2.9484	-11.95	-5.62622
81	ZINC20757502	0	-0.015	79.24	-1.41	-3.216	-9.1388	-10.05	-5.4821
82	ZINC20760260	0	0.597	92.05	-1.42	-3.464	-6.1207	-11.86	-6.39105
83	ZINC00927842	0	2.587	81.376	-0.64	-4.102	-8.9281	-4.154	-5.93347
84	ZINC14990781	0	1.786	84.227	-0.93	-2.801	-1.7858	-3.262	-6.45535
85	ZINC13511653	0	4.167	72.594	-0.01	-5.738	-7.0417	-2.651	-5.38878
86	ZINC15303567	0	2.532	83.12	-0.68	-5.635	-3.2904	-7.481	-6.2699
87	ZINC57608004	0	1.711	90.306	-1.05	-3.407	-9.4864	-4.157	-8.00755
88	ZINC20756673	0	0.047	79.24	-1.39	-3.119	-7.8451	-10.49	-5.85415
89	ZINC00928077	0	2.935	81.376	-0.53	-4.24	-9.1899	-5.284	-6.94729
90	ZINC63383785	0	0.359	89.805	-1.46	-3.174	-9.0284	-4.668	-4.57478
91	ZINC14540581	0	2.953	83.28	-0.55	-4.085	-8.2980	-6.421	-9.06778
92	ZINC14732374	0	2.603	70.726	-0.46	-4.195	-4.5155	-3.753	-9.93651
93	ZINC59286680	0	0.967	87.031	-1.23	-2.081	-2.6291	-14.39	-7.88
94	ZINC35804989	0	2.516	92.05	-0.83	-5.779	-5.3763	-7.744	-5.09289
95	ZINC16026352	0	2.516	92.05	-0.83	-5.807	-5.3763	-6.665	-5.68028
96	ZINC12571944	0	0.328	70.741	-1.17	-2.05	-5.8932	-9.493	-8.85582
97	ZINC35375166	0	1.093	76.07	-1.02	-3.827	-7.0719	-8.644	-4.42077

All numerical values are model-derived and unitless unless otherwise stated. logBB and logS represent predicted logarithmic values. HI: Human Intestinal; BBB: blood brain barrier; PPB: plasma protein binding
